# Wearable Signals for Diagnosing Attention-Deficit/Hyperactivity Disorder in Adolescents: A Feasibility Study

**DOI:** 10.1016/j.jaacop.2024.11.003

**Published:** 2024-11-25

**Authors:** Zhihan Jiang, Adrienne Y.L. Chan, Dawn Lum, Kirstie H.T.W. Wong, Janice C.N. Leung, Patrick Ip, David Coghill, Rosa S. Wong, Edith C.H. Ngai, Ian C.K. Wong

**Affiliations:** a Faculty of Engineering, The University of Hong Kong, Hong Kong SAR, China; b Groningen Research Institute of Pharmacy, Unit of Pharmacotherapy Epidemiology and Economics, University of Groningen, Groningen, The Netherlands; c Laboratory of Data Discovery for Health (D24H), The University of Hong Kong, Hong Kong SAR, China; d School of Pharmacy, University College London, London, United Kingdom; e Li Ka Shing Faculty of Medicine, The University of Hong Kong, Hong Kong SAR, China; f Hong Kong Children’s Hospital, Hong Kong SAR, China; g University of Melbourne, Melbourne, Victoria, Australia; h Aston School of Pharmacy, Aston University, Birmingham, United Kingdom

**Keywords:** activity monitoring, ADHD, machine learning, wearable devices

## Abstract

**Objective:**

Attention-deficit/hyperactivity disorder (ADHD) is a prevalent neurodevelopmental disorder in children and adolescents. Diagnoses of ADHD often rely on subjective ratings from parents and teachers. This study investigated the feasibility of using objective activity monitoring data collected through wearable activity trackers for ADHD diagnosis and monitoring in adolescents.

**Method:**

A longitudinal study was conducted involving Chinese adolescents ages 16 to 17 years. Data collected included objective measures (movement acceleration, heart rate, and sleep patterns from passive actigraphy) and subjective measures (parent and self-reported questionnaires). Machine learning models were developed using eXtreme Gradient Boosting (XGBoost) to compare various measures for ADHD classification. Model performance was evaluated using the area under the receiver operating characteristics curve (AUC). The SHapley Additive exPlanations (SHAP) method was used to analyze the importance of different measures on ADHD risk.

**Results:**

The study included 30 adolescents (17 with ADHD and 13 without ADHD). Machine learning models using solely objective measures achieved high predictability in classifying ADHD (AUC = 0.844) and ADHD medication status (AUC = 1.000). Models integrating both subjective and objective measures showed enhanced performance (AUC = 0.933). In this sample, key features for ADHD classification included irritability, sex, and quality-of-life indicators; key features for ADHD medication use classification included heart rate and physical activity intensity.

**Conclusion:**

Although the sample size was small, actigraphy-based monitoring provides a noninvasive and granular measurement of objective vital signs of adolescents. If validated in larger samples, the incorporation of objective measures is likely to enhance multidimensional assessment and diagnostic accuracy in adolescents with ADHD, supplementing existing diagnostic methods.

**Study preregistration information:**

A systematic review of anhedonia and amotivation in depression and cannabis use; https://www.crd.york.ac.uk/prospero/; CRD42023422438.

## Plain language summary

This study explores the feasibility of using commercial wrist-worn activity trackers in addition to parent- and self-report questionnaires to diagnose and monitor attention-deficit/hyperactivity disorder (ADHD) in Chinese adolescents. The data collected from 30 adolescents (17 with ADHD and 13 without ADHD) for 12 weeks, including movement patterns and heart rate, were used to develop machine learning models for ADHD identification and medication status classification. The experimental results combined and compared with traditional parent- and self-report questionnaires suggest that wearable trackers could offer an objective way to assess ADHD, potentially supplementing existing diagnostic methods and monitoring for this condition. If validated in larger samples, the incorporation of objective measures is likely to enhance multidimensional assessment and diagnostic accuracy in adolescents with ADHD.

Attention-deficit/hyperactivity disorder (ADHD) is the most common neurodevelopmental disorder in children, affecting approximately 6.1% of children in Hong Kong.^1^ ADHD can impact everyday activities, social interactions, and mental health, posing challenges for individuals, their families, and the community.^2^ Although clinical outcomes for children with ADHD can be measured through the routine use of symptom-based outcome measures, clinicians often report that they are too time-consuming for use within their busy clinics.^3^ Optimization of treatment relies on frequent monitoring by parents, teachers, and clinicians. The retrospective nature of monitoring and reporting both is time-intensive and may lead to recall and reporter bias. Due to the dependence on third parties, often optimizing treatment is unfeasible in busy school and clinic environments. Thus, there are currently no biomarkers or other objective measures available to identify ADHD or monitor treatment response.

At the present time, diagnosis and measurement of treatment response in ADHD rely on retrospective, subjective accounts, predominantly parent and teacher reports,^4^ which can be challenging due to the high heterogeneity in the presentation of ADHD, similarities to other mental health disorders, and high rates of comorbidities. Objective measures such as actigraphy, a passive measure of rest/activity cycles, have been extensively used in research to measure activity and sleep, which may prove useful in the diagnosis and monitoring of treatment response in ADHD. Actigraphy provides a naturalistic and objective way to monitor activity across the day and over extended periods of time, potentially offering a valuable alternative to subjective measures.

Although existing studies have examined the correlation between actigraphy characteristics and ADHD, there is a lack of direct evidence and application on the identification and monitoring of ADHD through use of commercial activity trackers. As Fitbit devices are one of the most popular consumer-oriented wearable devices to monitor body activity, they have the potential to be an accessible tool used by clinicians to diagnose and monitor ADHD.^5^ Moreover, machine learning models such as XGBoost^6^ have shown outstanding performance in handling wearable sensing data. Furthermore, the SHapley Additive exPlanations (SHAP) method, which is a game theoretic approach measuring the feature importance and interpreting the output of machine learning models, is powerful and has achieved great success in various domains including health care. Through analyzing the SHAP values, we can measure the contribution of each feature to the prediction results in a quantitative way.

This study explored whether using machine learning and analysis of moment-to-moment activity monitoring data collected through the Fitbit activity tracker is a feasible objective clinical tool to aid the diagnosis and monitoring of adolescents with ADHD. By explaining the machine learning models, this study analyzed the importance and influence of different data on ADHD diagnosis and monitoring. While the results of this study should be viewed as preliminary due to the limited sample size, we believe it can be a crucial first step in research development involving commercial activity trackers and advanced machine learning techniques in Chinese adolescents, providing initial insights that guide larger and more comprehensive studies.

## Method

### Study Design and Data Source

This longitudinal study investigated the feasibility of activity monitoring using Fitbit (Google LLC, Mountain View, California) activity tracking devices^7^ in everyday clinical practice in adolescents with ADHD. The manufacturer of Fitbit has ensured that it complies with all applicable Health Insurance Portability and Accountability Act requirements.^8^

### Participants

Participants recruited for the study were Chinese adolescents in Hong Kong ages 16 to 17. They were selected from a larger cohort recruited between 2011 and 2012 from 20 kindergartens, as reported in previous publications.^9^, ^10^, ^11^ The cohort included 820 children (between 2011 and 2012), with 6.59% of them having a recorded diagnosis of ADHD identified from the Hong Kong Clinical Data Analysis Reporting System (CDARS). Within the cohort, 92.56% of children were typically developing, and 0.85% of children had other mental health problems. A detailed description of the recruitment process through convenience sampling, which occurred between September 1 and 7, 2021, is available in Supplements 1 and Supplement 2, available online. A series of inclusion and exclusion criteria were then used to select participants from the cohort for this study. The inclusion criteria for the participants included being between the ages of 12 and 17 years at the time of enrollment, owning and being able to use a smartphone, and attending school. Participants in the ADHD group also must have had a recorded diagnosis in CDARS. Participants were excluded if they had an urgent mental health referral to a hospital (eg, suicidality or psychosis), had a major illness or disability including neurological and movement disorders (eg, epilepsy or Tourette’s disorder) that would confound the actigraphy data, previously received a diagnosis of autism spectrum disorder from a health care professional, had an intellectual disability (ie, IQ <70), or could not understand Chinese or English. The group without ADHD comprised both typically developing children and children with other mental health problems. Finally, we recruited 30 participants (17 participants with ADHD) in this study for further data collection.

### Data Collection

Participants provided written consent and completed self-report questionnaires on tablet computers via Qualtrics (Seattle, Washington) every 4 weeks over a 12-week observation period without affecting their clinical care. They reported Fitbit removal of over an hour and ADHD medication usage weekly via WhatsApp (Meta, Menlo Park, California). Participants received HK$100 per week and were allowed to keep the Fitbit wearable device after the study. The study protocol was approved by the Institutional Review Board of the University of Hong Kong/Hospital Authority Hong Kong West Cluster.

Diagnoses and treatment information were obtained from CDARS electronic medical records. CDARS is an electronic health database for clinical audit and research purposes that is managed by the Hong Kong Hospital Authority. The data recorded in CDARS include demographics, diagnosis, hospitalizations, causes, and dates of death. Activity data were collected using Fitbits and smartphones. We measured 2 different kinds of activity: movement measured by acceleration (physical body movement) and physiological activity measured by heart rate and sleep. Table 1 summarizes the measurement instruments (ie, Fitbit or questionnaires), activity types, and data collection intervals. Passive Fitbit outcomes were examined in conjunction with the participants’ daily schedules to provide a context for the recorded activity, sleep, and heart rate data. Other outcomes were assessed by parent-reported or self-reported questionnaires on ADHD symptoms. The parent-reported questionnaire used to assess ADHD was the Strengths and Weakness of ADHD Symptoms and Normal Behavior Scale (SWAN).^12^ The self-reported questionnaires included the Center for Epidemiologic Studies Depression Scale (CES-D),^13^ Teenage Executive Functioning Inventory Scale (TEXI),^14^ Affective Reactivity Index (ARI),^15^ and Pediatric Quality of Life Scale (PedsQL),^16^ which measure depressive symptoms, executive functioning, irritability symptoms, and quality of life at baseline and during follow-up. All participants were previously screened for ADHD using the SWAN ADHD module and diagnosed by clinicians who distinguished participants into 1 of the 3 ADHD subtypes: inattentive, hyperactive, and combined inattentive/hyperactive. We also collected participants’ sex, ethnicity, education level, comorbidities, and ADHD treatments.

**Table 1:** Summary of Measures Included in Study

Measure	Description	Time intervals	Method
Accelerometry	Physical activity including daily calories burned, daily walking distance, daily floors, daily sedentary minutes, daily light active minutes, daily fair active minutes, daily very active minutes, daily activity calories, hourly steps	Continuous	Objective measure by Fitbit Charge 4 wristband
Heart rate	Mean heart rate, maximal heart rate, resting heart rate	Continuous	Objective measure
Sleep	Duration, stage, and quality	Continuous	Objective measure
SWAN Rating Scale	Parent questionnaire assessing symptoms of ADHD and determining ADHD subtypes (hyperactive-impulsive, inattentive, and combined type)	N/A	Parent-reported subjective measure
Depressive symptoms	CES-D^12^^,^^13^—a 20-item questionnaire assessing depressive symptoms over the past week	Baseline, wk 4, 8, 12	Self-reported subjective measure
Executive functioning	TEXI^14^—a 20-item scale measuring executive functioning	Baseline, wk 4, 8, 12	Self-reported subjective measure
Irritability	ARI^15^—a 6-item validated scale measuring irritability	Baseline, wk 4, 8, 12	Self-reported subjective measure
Quality of life	PedsQL 4.0^16^—a 23-item validated measure for children ages 2-18 y; provides total, physical, and psychosocial health summary scores, with higher scores indicating better health-related quality of life	Baseline, wk 4, 8, 12	Self-reported subjective measure
Sex	N/A	Baseline	Self-report objective measure

Note: ADHD = attention-deficit/hyperactivity disorder; ARI = Affective Reactivity Index; CES-D = Center for Epidemiologic Studies Depression Scale; N/A = not applicable; PedsQL = Pediatric Quality of Life Scale; SWAN = Strengths and Weakness of ADHD Symptoms and Normal Behavior Scale; TEXI = Teenage Executive Functioning Inventory Scale.

### Data Processing

The data collected were grouped into self-reported subjective features, parent-reported subjective features, and objective features. Specifically, the self-reported subjective features included TEXI working memory score, TEXI inhibition score and total score of TEXI; PedsQL physical health score and PedsQL psychosocial health score; and total scores of PedsQL, ARI, and CES-D. The parent-reported subjective features included the SWAN hyperactivity score, SWAN inattentiveness score, and total score of SWAN. The objective features included sex, daily features (sleep duration, calories burned, walking distance, floors, sedentary minutes, light active minutes, fair active minutes, very active minutes, activity calories), and hourly features (heart rate and step counts), calculated after the removal of Fitbit taken off time. Specifically, the hourly step counts were also calculated after the removal of sleep time. Additionally, only heart rate data with confidence scores over 2 out of 3, as recorded by Fitbit, were included in the final analysis. The confidence score was directly measured by Fitbit and calculated between 0 and 3. A higher score indicated a greater confidence in the accuracy of the Fitbit data. A complete case analysis approach was adopted where the unit count was by days or hours. Participant days/hours with missing data were discarded. The features were determined by aggregating and averaging all participant days/hours contributed for each participant. While these measures are labeled parent-reported and self-reported for easier reporting in the article, we did not intend to compare self-report and parent-report as different instruments were used.

### Descriptive Statistics

The baseline characteristics of participants were summarized by ADHD status. Summary statistics from poststudy surveys of participants were used to assess the acceptance of using Fitbit in everyday lives.

### Machine Learning Algorithm for ADHD and ADHD Medication Status Classification

We aimed to explore the effectiveness of machine learning models with different features in identifying participants with ADHD and classifying the medication status of ADHD. To this end, we first trained the machine learning models for ADHD identification based on XGBoost.^6^ XGboost is an ensemble machine learning algorithm based on gradient-boosted decision trees. It has achieved outstanding performance in various domains due to its low computational cost, high efficiency, and robustness to overfitting.^17^

Based on the self-reported subjective, parent-reported subjective, and objective features collected (detailed in Table 1), we trained models with different combinations of these features to explore the optimal set of features required for ADHD diagnosis and ADHD medication use classification. We compared the effectiveness of self-reported subjective and objective features by training models with both self-reported subjective and objective features (model 1), self-reported subjective features (model 2), and objective features (model 3). We trained a model relying on observer reports with the parent-reported subjective features (model 4). We also trained a model with all features (ie, self-reported subjective, parent-reported subjective, and objective features) (model 5). Based on these models, we evaluate the performance of the machine learning models in classifying ADHD vs without ADHD and medicated vs unmedicated people with ADHD.

The models were trained on 80% of the data and tested using the remaining 20%. For each model, the parameters were determined by grid search and validated through 5-fold cross-validation on the training set. In every iteration, the dataset was divided into 5 nearly equal parts. The model was trained and tested for 5 iterations, each iteration using a different fold as the test set and the remaining 4 parts as the training set. Averaging results over 5 folds reduces variability and ensures robust model evaluation, which is particularly beneficial for small sample sizes. The sets of parameters that achieved the best performance were selected for further analysis. The evaluation metric used was the area under the receiver operating characteristic curve (AUC) obtained by plotting the true-positive rate (sensitivity) against the false-positive rate (1 − specificity) at various threshold settings.

Then, based on the optimal probability threshold leading to the best predictive performance of the selected algorithm, values of accuracy, sensitivity, specificity, positive predictive value (PPV), and negative predictive value (NPV) on the optimal threshold were calculated for ADHD, without ADHD, medicated ADHD, and unmedicated ADHD groups. The definitions and calculations of each metric are described in Supplement 3, available online. In summary, we compared the accuracy, sensitivity, specificity, PPV, NPV, and AUC under the settings (models 1-5) described above. All results are averaged values over 5-fold cross-validation. CIs are estimated based on the Wilson score interval for accuracy, sensitivity, specificity, PPV, and NPV and bootstrapping on the cross-validation scores for AUC.

### Feature Importance Analysis for Model Interpretability

We conducted SHAP analyses to measure the feature importance and to allow for the interpretation of the predicted outcomes of machine learning models. SHAP is a game theoretic method that computes the contribution of each feature in a machine learning model. It can be implemented efficiently, especially for tree-based models. In this study, SHAP analysis was used to understand the global impact of input features on the overall model. The SHAP values also provide the capability to explore the local feature effects, which illustrates the impact of input features on individual predictions. This was later used to present the 3 most influential features for each model prediction. To explore the correlation between the output and features, we visualized and listed important features and their SHAP values of our machine learning models. A positive SHAP value increases the probability of the participant being classified as a person with ADHD. Through analyzing the SHAP values of features for each model, the important features can be identified, and the correlation between important features and the output can provide us with insights into the factors correlated with ADHD. Furthermore, we normalized the shape values by the following equation:

$$\bar{SHAP\left(i\right)}=\frac{SHAP\left(i\right)}{\sum_{j=1}^{N}SHAP\left(j\right)}\text{,}$$

where *SHAP(i)* is the SHAP value of feature *i*, and $\sum_{j=1}^{N}SHAP\left(j\right)$ is the sum of SHAP values of all input features.^18^ Further details on calculating SHAP values can be found in Supplement 3, available online.

All analyses were performed using Python software version 3.8.2 (Python Software Foundation, Wilmington, Delaware), R software 4.1.1 (R Foundation for Statistical Computing, Vienna, Austria), and SAS software version 9.4 (SAS Institute Inc., Cary, North Carolina).

## Results

### Statistical Description of Participants

All participants were of ethnic Chinese descent; 1 participant also had Vietnamese heritage. Comorbidities were present in 5 participants with ADHD and 1 typically developing participant. Furthermore, 29 participants were in secondary education and 1 participant was in postsecondary education. Among the 30 participants, 17 had a diagnosis of ADHD; 13 participants with ADHD reported a history of ADHD medication treatment, but only 12 participants with ADHD took ADHD medication during the study period, 1 of whom took medication only for test or examination days (twice during the study period). The sociodemographic characteristics of participants are summarized in Table 2. The mean (SD) number of days contributed by each participant was 85.83 (9.89) days.

**Table 2:** Sociodemographic Characteristics of Participants

Characteristic	ADHD (n = 17)		Without ADHD (n = 13)	
	n	(%)	n	(%)
Sex, male	13	(76)	3	(23)
Ethnicity				
Chinese	17		12	
Chinese/Asian mixed race/ethnicity	0		1	
Education level				
Secondary	16		13	
Postsecondary (associate degree/diploma)	1		0	
Comorbidities				
Bulimia	1		0	
Dyslexia	1		0	
Insomnia	1		0	
Depression	1		0	
Arthritis	1		0	
Anorexia nervosa	0		1	
ADHD treatment received	15		N/A	
Type of treatment				
Medication^a^				
Methylphenidate (Ritalin)	5		N/A	
Methylphenidate (Concerta)	8		N/A	
Behavioral therapy	4		N/A	
Other	1		N/A	
Frequency of ADHD medication taken per day				
Once	8		N/A	
Twice	2		N/A	
3 or more times	1		N/A	
Only taken on test days	1		N/A	
Once to twice	1		N/A	

Note: ADHD = attention-deficit/hyperactivity disorder; N/A = not applicable.

a In Hong Kong, only Ritalin and Concerta are licensed for use and available by prescription.

### Statistical Description of Features

**Figure 1:**
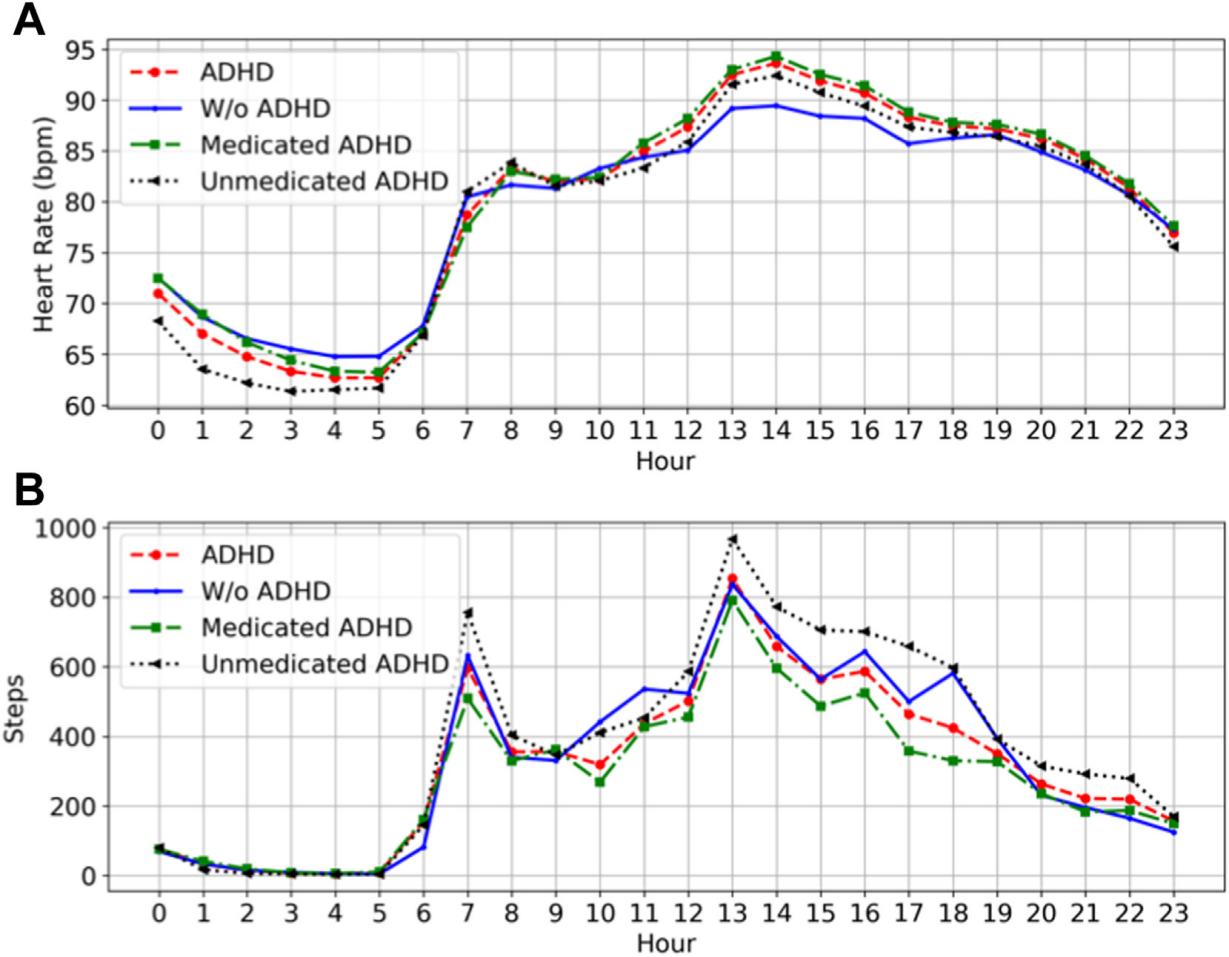
Mean Values of 24-Hour Patterns of Different Groups ***Note:****Attention-deficit/hyperactivity disorder (ADHD) and without ADHD (W/o ADHD) lines are the mean of all participants in the ADHD and without ADHD groups, respectively. The medicated and unmedicated lines are the mean of medicated ADHD and unmedicated ADHD participants, respectively. (A) Heart rate patterns. (B) Step patter*ns. *bpm = beats per minute.*

Mean scores of subjective, objective, and parent-reported subjective measures (SWAN scores) of the different groups are shown in Table 3, Table 4, and 5, respectively. For hourly objective features (including heart rate and step counts), we calculated the 24-hour patterns for each participant and obtained the mean values of the 24-hour patterns of the participants in each group (Figure 1). Figure 1A, showing heart rate comparisons of different groups, found that the heart rate of the ADHD-diagnosed group was lower than the without ADHD group during hours 1 to 7, but higher for the majority of hours 8 to 23. Also, the medicated participants generally had a higher heart rate. Figure 1B showed that the step count of ADHD-diagnosed and medicated participants was generally lower than participants without ADHD and unmedicated participants, respectively, during most of the day.

**Table 3:** Scores of Subjective Measures of Different Groups

Group	TEXI						PedsQL						ARI		CES-D	
	Working memory		Inhibition		Total score		Physical health		Psychosocial health		Total score		Total score		Total score	
	Mean	(SD)	Mean	(SD)	Mean	(SD)	Mean	(SD)	Mean	(SD)	Mean	(SD)	Mean	(SD)	Mean	(SD)
ADHD	24.912	(6.171)	13.441	(3.603)	38.353	(9.125)	83.502	(10.622)	71.716	(13.620)	75.815	(11.825)	2.956	(2.090)	14.338	(8.282)
Without ADHD	19.635	(5.673)	12.077	(3.621)	31.712	(8.611)	83.413	(8.671)	77.019	(9.221)	79.243	(8.355)	0.808	(0.843)	14.327	(8.822)
Medicated ADHD	24.477	(6.694)	14.318	(3.258)	40.795	(9.519)	81.392	(10.660)	70.0	(11.492)	73.962	(10.813)	3.0	(2.630)	15.364	(5.132)
Unmedicated ADHD	22.042	(5.558)	11.833	(3.614)	33.875	(8.326)	87.370	(10.481)	74.861	(14.886)	79.212	(12.432)	2.875	(1.877)	12.458	(9.659)

Note: ADHD = attention-deficit/hyperactivity disorder; ARI = Affective Reactivity Index; CES-D = Center for Epidemiologic Studies Depression Scale; PedsQL = Pediatric Quality of Life Scale; TEXI = Teenage Executive Functioning Inventory Scale.

**Table 4:** Scores of Objective Measures of Different Groups

Group	Sleep duration		Calories burned		Walking distance		Floors		Sedentary minutes		Light active minutes		Fair active minutes		Very active minutes		Activity calories	
	Mean	(SD)	Mean	(SD)	Mean	(SD)	Mean	(SD)	Mean	(SD)	Mean	(SD)	Mean	(SD)	Mean	(SD)	Mean	(SD)
ADHD	433.114	(56.411)	2,375.294	(396.660)	5.088	(1.641)	13.465	(5.067)	555.122	(255.326)	232.820	(41.183)	26.928	(5.804)	18.348	(8.445)	1,093.312	(258.144)
Without ADHD	409.688	(46.304)	2,209.065	(318.260)	5.267	(2.035)	12.876	(6.041)	555.944	(222.307)	213.209	(42.339)	21.324	(15.256)	12.389	(11.438)	922.446	(303.199)
Medicated ADHD	435.250	(55.014)	2,382.590	(370.276)	4.412	(1.812)	12.783	(7.302)	529.305	(205.067)	231.869	(45.725)	26.516	(18.770)	15.635	(11.713)	1,080.768	(359.986)
Unmedicated ADHD	429.197	(27.922)	2,361.917	(222.675)	6.329	(1.956)	14.714	(2.689)	602.453	(264.360)	234.563	(39.359)	27.684	(6.252)	23.322	(9.919)	1,116.310	(184.429)

Note: ADHD = attention-deficit/hyperactivity disorder.

**Table 5:** Scores of Parent-Reported Subjective Measures of Different Groups

Group	Hyperactivity score		Inattentive score		Combined score	
	Mean	(SD)	Mean	(SD)	Mean	(SD)
ADHD	0.196	(0.826)	0.611	(1.008)	0.369	(0.948)
Without ADHD	−1.282	(0.998)	−0.607	(0.963)	−0.944	(0.954)
Medicated ADHD	0.030	(0.935)	0.348	(1.101)	0.136 (1.062)	(1.062)
Unmedicated ADHD	0.500	(0.515)	1.093 (0.634)	(0.634)	0.796	(0.530)

Note: ADHD = attention-deficit/hyperactivity disorder.

### ADHD Status Classification Performance

#### Classification Between ADHD and Without ADHD

As shown in Table 6, when comparing models 1 through 4, we found that model 1 using both self-reported subjective and objective measures achieved the best performance on all metrics (accuracy = 0.967 [95% CI 0.902, 1.000], sensitivity = 1.0 [95% CI 0.772, 1.000], specificity = 0.941 [95% CI 0.730, 0.990], PPV = 0.929 [95% CI 0.685, 0.987], NPV = 1.0 [95% CI 0.806, 1.000], AUC = 0.933 [95% CI 0.789, 1.000]), indicating that a combination of self-reported subjective and objective features can improve ADHD identification performance. Model 3 using only objective measures achieved the best performance on specificity (0.941 [95% CI 0.730, 0.990]) and the second best on accuracy (0.933 [95% CI 0.844, 1.000]), sensitivity (0.923 [95% CI 0.667, 0.986]), PPV (0.923 [95% CI 0.667, 0.986]), and NPV (0.941 [95% CI 0.730, 0.990]). Model 4 using parent-reported features achieved the second best performance on sensitivity (0.923 [95% CI 0.667, 0.986]) and AUC (0.867 [95% CI 0.667, 1.000]). We found that although a combination of self-reported subjective, objective, and parent-reported subjective features could achieve satisfactory performance (AUC = 0.933 [95% CI 0.789, 1.000]), it does not outperform the model based on self-reported subjective and objective features (AUC = 0.933 [95% CI 0.789, 1.000]), indicating that the self-reported subjective and objective measures are sufficient for ADHD identification.

**Table 6:** Performance of 4 Machine Learning Models With Different Input Features and Clinical Cutoffs

Groups	Input features	Results											
		Accuracy	(95% CI)	Sensitivity	(95% CI)	Specificity	(95% CI)	PPV	(95% CI)	NPV	(95% CI)	AUC	(95% CI)
ADHD (n = 17) and Without ADHD (n = 13)	Model 1	0.967	(0.902, 1.000)	1.000	(0.772, 1.000)	0.941	(0.730, 0.990)	0.929	(0.685, 0.987)	1.000	(0.806, 1.000)	0.933	(0.789, 1.000)
	Model 2	0.867	(0.745, 0.988)	0.846	(0.578, 0.957)	0.882	(0.657, 0.967)	0.846	(0.578, 0.957)	0.882	(0.657, 0.967)	0.856	(0.678, 1.000)
	Model 3	0.933	(0.844, 1.000)	0.923	(0.667, 0.986)	0.941	(0.730, 0.990)	0.923	(0.667, 0.986)	0.941	(0.730, 0.99)	0.844	(0.678, 1.000)
	Model 4	0.833	(0.700, 0.967)	0.923	(0.667, 0.986)	0.765	(0.527, 0.904)	0.750	(0.505, 0.898)	0.929	(0.685, 0.987)	0.867	(0.667, 1.000)
	Model 5	0.933	(0.844, 1.000)	0.923	(0.667, 0.986)	0.941	(0.730, 0.990)	0.923	(0.667, 0.986)	0.941	(0.730, 0.990)	0.933	(0.789, 1.000)
	Parent-reported subjective feature (research cutoff for SWAN)	0.767	(0.616, 0.918)	0.647	(0.413, 0.827)	0.923	(0.667, 0.986)	0.917	(0.646, 0.985)	0.667	(0.437, 0.837)	N/A	(N/A)
	Top 17 features of model 1	0.967	(0.902, 1.000)	1.000	(0.772, 1.000)	0.941	(0.730, 0.990)	0.929	(0.685, 0.987)	1.000	(0.806, 1.000)	0.911	(0.789, 1.000)
	Top 11 features of model 5	0.933	(0.844, 1.000)	0.923	(0.667, 0.986)	0.941	(0.730, 0.990)	0.923	(0.667, 0.986)	0.941	(0.730, 0.990)	0.900	(0.728, 1.000)
Medicated ADHD (n = 11) and unmedicated ADHD (n = 6)	Model 1	0.941	(0.829, 1.000)	1.000	(0.610, 1.000)	0.909	(0.623, 0.984)	0.857	(0.487, 0.974)	1.000	(0.722, 1.000)	1.000	(1.000, 1.000)
	Model 2	0.882	(0.729, 1.000)	1.000	(0.610, 1.000)	0.818	(0.523, 0.949)	0.750	(0.409, 0.929)	1.000	(0.701, 1.000)	0.900	(0.550, 1.000)
	Model 3	0.941	(0.829, 1.000)	1.000	(0.610, 1.000)	0.909	(0.623, 0.984)	0.857	(0.487, 0.974)	1.000	(0.722, 1.000)	1.000	(1.000, 1.000)
	Model 4	0.353	(0.126, 0.580)	1.000	(0.610, 1.000)	0.000	(0.000, 0.259)	0.353	(0.173, 0.587)	N/A	(N/A)	0.500	(0.200, 0.667)
	Model 5	0.941	(0.829, 1.000)	1.000	(0.610, 1.000)	0.909	(0.623, 0.984)	0.857	(0.487, 0.974)	1.000	(0.722, 1.000)	1.000	(1.000, 1.000)

Note: Model 1: both self-reported subjective and objective features; model 2: self-reported subjective features; model 3: objective features; model 4: parent-reported subjective (SWAN) features; model 5: self-reported subjective, parent-reported subjective, and objective features; and research cutoffs on parent-reported subjective features. All results are averaged over 5-fold cross-validation. CIs are estimated based on the Wilson score interval for accuracy, sensitivity, specificity, PPV, and NPV and bootstrapping on the cross-validation scores for AUC. ADHD = attention-deficit/hyperactivity disorder; AUC = area under the receiver operating characteristics curve; N/A = not available; NPV = negative predictive value; PPV = positive predictive value; SWAN = Strengths and Weakness of ADHD Symptoms and Normal Behavior Scale.

#### Classification Between Medicated and Unmedicated ADHD

Although there were 12 participants who took medication during the study period, 1 participant took medicine only twice. Therefore, we calculated the features after removing the time with medication effectiveness and regarded this participant as unmedicated ADHD. In this way, there were 11 medicated participants with ADHD and 6 unmedicated participants with ADHD. Table 6 shows that the model 4 trained on SWAN scores did not accurately predict the classification (AUC = 0.500 [95% CI 0.200, 0.667]). Model 1 with both self-reported subjective and objective features (AUC = 1.000 [95% CI 1.000, 1.000]); model 3 with objective features only (AUC = 1.000 [95% CI 1.000, 1.000]); and model 5 with self-reported subjective, objective, and parent-report (SWAN) features (AUC = 1.000 [95% CI 1.000, 1.000]) achieved the best performance on all metrics, indicating that the objective features are enough to achieve outstanding performance on classification between medicated ADHD and unmedicated ADHD. Model 2 using only self-reported subjective features also achieved higher predictability compared with SWAN scores (AUC = 0.900 [95% CI 0.550, 1.000]).

### Feature Importance Analysis

**Figure 2:**
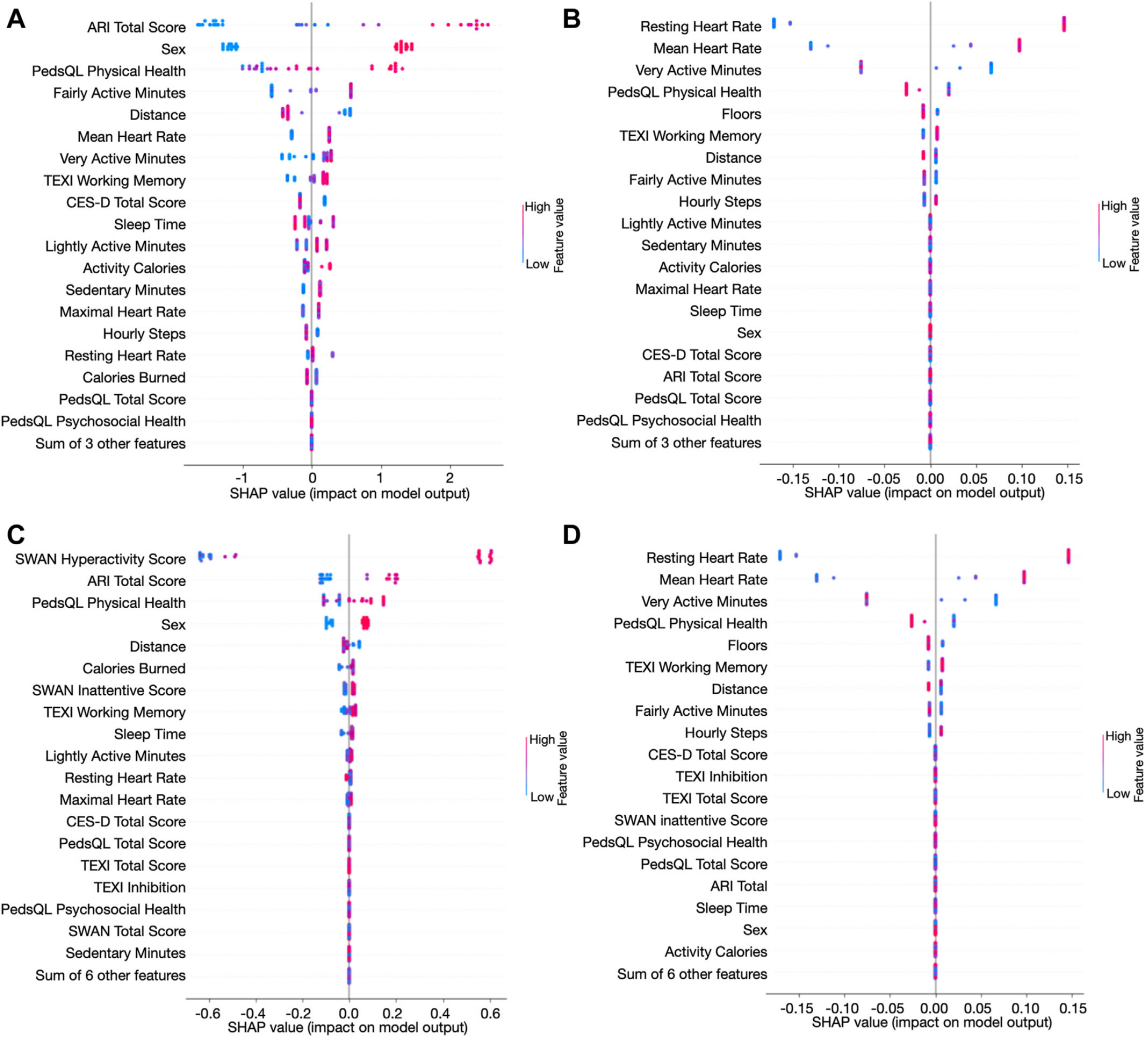
SHapley Additive exPlanations (SHAP) Values of Various Input Features for Machine Learning Models ***Note:****The features are ranked by importance (most important is at the top). Red color means higher feature values, and blue color means lower feature values. A positive SHAP value means the feature increases the model output, whereas a negative SHAP value means the feature decreases the model output. (A) SHAP values based on the model trained on both objective and self-reported subjective measures for classifying attention-deficit/hyperactivity disorder (ADHD) and without ADHD. (B) SHAP values based on the model trained on both objective and self-reported subjective measures for classifying medicated ADHD and unmedicated ADHD. (C) SHAP values based on the model trained on objective, self-reported subjective, and parent-reported features for classifying ADHD and without ADHD. (D) SHAP values based on the model trained on objective, self-reported subjective, and parent-reported subjective features for classifying medicated ADHD and unmedicated ADHD. ARI = Affective Reactivity Index; CES-D = Center for Epidemiologic Studies Depression Scale; PedsQL = Pediatric Quality of Life Scale; SWAN = Strengths and Weakness of ADHD Symptoms and Normal Behavior Scale; TEXI = Teenage Executive Functioning Inventory Scale.*

For model 1, which performed best on both tasks, SHAP values revealed the top 3 most important features: ARI total score, sex, and PedsQL physical health (Figure 2A). ARI measures irritability. Higher levels of irritability, male sex, and lower physical health from PedsQL (lower scores) increased the probability of being predicted as participants with ADHD. Other features such as daily walking distance, mean heart rate, daily very active minutes, TEXI working memory score, and CES-D total score also had strong associations with ADHD prediction. The monotonic association between other features and ADHD probability was weak or negligible.

Figure 2B shows the SHAP values of the model achieving the best performance on classifying medicated and unmedicated ADHD. Resting heart rate, mean heart rate, and very active minutes were the top 3 most important features. The results showed that higher resting heart rates and higher mean heart rates increased the probability of being predicted as medicated ADHD. Also, a lower PedsQL physical health score decreased the probability of being predicted as unmedicated ADHD. The feature importance analysis results showed that resting heart rate is a critical feature for identifying medicated ADHD.

We further explored the SHAP values of model 5 trained on all features to study the influence of SWAN features compared with other features. The combination of self-reported subjective and objective features had a stronger predictability power (Figure 2A), as seen in the greater range of SHAP values, than using combined self-reported subjective, parent-reported subjective, and objective features (Figure 2C). However, the SWAN hyperactivity value was the highest in Figure 2C, showing that SWAN features are still effective in predicting ADHD. Higher inattentive SWAN scores also increased the probability of being predicted as ADHD, consistent with the assessment of the SWAN scale.

After normalizing the SHAP values of models 1 and 5, for model 5 for classifying ADHD and without ADHD, the cumulative normalized SHAP values of the top 11 most important features account for more than 99% of the sum of all normalized SHAP values, whereas for model 1, the top 17 most important features are required. We pruned the less important features and used the top 11 and 17 most important features as the input of model 5 and model 1, respectively. The results are shown in Table 6. In this way, we reduced the model complexity while maintaining satisfactory performance. Fewer features were required to achieve good performance after incorporating SWAN features, but the parent-reported subjective features had a significantly higher impact on the model output than other features, making the model ignore some other critical features, such as mean heart rate, very active minutes, and CES-D total score. Thus, the performance of model 5 was slightly lower than that of model 1 after feature pruning.

Figure 2D showed the SHAP values of input features of model 5 trained on objective, self-reported subjective, and parent-reported features for medicated ADHD and unmedicated ADHD classification. Parent-reported scores have little impact on the model output, and the feature importance is similar to that shown in Figure 2B, indicating the small influence of parent-reported SWAN features on classifying ADHD medication status. The detailed rankings of features based on SHAP values from the model trained on different features for ADHD identification and classification can be found in Table S2 in Supplement 4, available online.

### Evaluation of Impact on Daily Lives

Of the 30 participants, 27 (90%) completed the poststudy acceptance questionnaire. (13 in the ADHD group and 14 in the without ADHD group). The survey includes 5 questions regarding study instruction clarity, impact on lifestyle outside of school, impact on school life, willingness to continue using Fitbit, and problems while wearing Fitbit (detailed in Table S1 in Supplement 4, available online).

Most participants acknowledged the clarity of our study, with 84% in the ADHD group and nearly 100% in the without ADHD group selecting “slightly” or “very clearly.” Only 3 participants had a neutral attitude. Regarding the ease of reporting data, participants reported that “reminders from researchers to sync Fitbit data,” “positive attitude of researchers,” “easy and convenient contact methods (WhatsApp),” and “responsive feedback from researchers” were facilitating factors to the simplicity of submitting data in the study.

All participants claimed that the wearable devices (ie, Fitbit) had no impact or positive impact on their lifestyle outside of school with reasons such as “the metrics from Fitbit could encourage them to do more exercise,” “document stress,” and “feeling fulfilled after working out.” Regarding the impact of wearable devices on school life, 3 participants with ADHD and 1 participant without ADHD reported a somewhat negative impact with reasons such as “distracting during classes” and “the school does not allow wearing of Fitbit, they would have to hide.” Other participants reported positive impacts such as convenience for timekeeping during examinations and tests, “for reading the time,” and “detect if they feel stressed.” Regarding the problems they met when using Fitbit (a multiple-selection question), the most frequent problem is “easy to forget to re-wear,” and the second is “uncomfortable.” Some participants reported “the buzzing from Fitbit could be distracting,” and many participants reported “they often forget to put the Fitbit back on after removing” (eg, shower) and discomfort such as irritation on the skin as problems. These results indicate the research opportunity of making wearable devices more engaged in users’ daily lives and more comfortable to wear. Regarding the willingness to continue using Fitbit, some participants selected “probably,” “might,” or “might not,” and more participants selected “probably yes” and “definitely yes” for reasons such as “wearing Fitbit improves their health” and “to learn more about their sleep and activity habits.” As this question is specific to Fitbit Charge 4 devices, it cannot represent the acceptance of all wearable devices. The willingness to continuously wear devices can be influenced by various factors such as the product appearance, function, and comfort. Generally, the participants have a positive attitude toward using wearable devices for routine monitoring, indicating high acceptance from the user side.

## Discussion

The experimental results demonstrate that wearable devices (eg, actigraphy) coupled with machine learning techniques are viable, valid, and reliable objective clinical tools to aid the diagnosis and monitoring of adolescents with ADHD. These tools show enhanced performance when integrating both subjective and objective measures. Significant features identified include the ARI total score, sex, and PedsQL physical health score for distinguishing ADHD from without ADHD, and resting heart rate, mean heart rate, and very active minutes for classifying medicated and unmedicated ADHD.

Irritability is a common symptom often observed in people with ADHD due to the impact of the disorder on emotional regulation.^19^ ADHD can contribute to difficulties in managing stress and frustration. This emotional aspect can affect behavior and attention, characteristics central to ADHD diagnosis. Also, research has consistently shown that ADHD is more frequently diagnosed in male youth compared with female youth.^20^ This could be partly due to the externalizing behaviors more commonly exhibited by male youth, which are easier to identify as symptoms of ADHD in traditional diagnostic settings. Physical health, as measured by the PedsQL physical functioning scale, is typically lower in youth with ADHD due to issues such as motor coordination difficulties, impulsivity, and hyperactivity.^21^ Additionally, comorbid conditions such as obesity^22^ and sleep disorders,^23^ alongside side effects from ADHD medications,^24^^,^^25^ can affect overall activity levels. Contrary to what might be expected, highly active minutes or distance did not correlate as strongly with ADHD diagnosis in our study. This could be because there were more inattentive presentations than hyperactive presentations in our study. The manifestation of ADHD can change during adolescence: hyperactivity may decrease, and problems with attention, impulsivity, and executive functioning might become more prominent. It also suggests that although hyperactivity is a hallmark of ADHD, the quantifiable extremities of physical activity might not directly align with the diagnostic criteria or might be influenced by other compensatory behaviors or interventions.

The findings highlight the significance of combining subjective and objective measures in ADHD identification, with objective measures proving especially effective in ADHD medication status classification, indicating that wearable technology can be a promising complement to existing diagnostic protocols. Specifically, the machine learning models outperform directly using clinical cutoffs of the SWAN scale. This may be because many existing cutoffs are proposed based on the groups included in the studies, hindering the generalisability of the cutoffs. Although the machine learning models are also trained based on data collected from the studied groups, compared with cutoffs, they are easier to adapt to other groups by transfer learning and are more flexible. Besides, directly using the cutoffs cannot reveal the factors increasing the ADHD risk, while by interpreting the machine models, the importance and influence of various factors can be estimated.

As depicted in Figure 1B in our study, the ADHD group exhibited lower step counts than the without ADHD group for most of the day. This may result from less engagement in continuous physical activities of participants with ADHD with inattentive presentations,^26^ hyperfocus on sedentary activities,^27^ and decreasing participation in physically active social activities due to social interaction difficulties and emotional regulation challenges.^28^^,^^29^ Despite the idea that hyperactivity might suggest more movement, the impulsive traits of ADHD can cause quick activity changes, preventing sustained engagement for accumulating significant steps.^30^

The findings of this feasibility study provide initial insights into the behavioral, biological, psychological, and social factors involved in ADHD. However, we caution that the small subgroup sizes may limit the generalizability and reliability of the results. These results should be viewed as preliminary necessitating further validation in larger and more representative samples.

In contrast to previous work, our study investigated the feasibility of using actigraphy for ADHD diagnosis and monitoring directly with explainable machine learning. Three meta-analyses of studies using actigraphy including 90 studies (n = 2890 children with ADHD and n = 3528 healthy controls) consistently found that children with ADHD have a higher daytime mean activity and altered sleep patterns.^31^, ^32^, ^33^ These existing studies focused on statistical differences in actigraphy between ADHD and without ADHD groups^34^ without directly using actigraphy for ADHD diagnosis and monitoring. Some studies^35^^,^^36^ built ADHD prediction models using actigraphy data. However, they relied on research or medical devices to provide raw fine-grained sensor data, which often remain inaccessible for many commercial activity trackers due to the high storage and transmission costs. Instead, higher-level features, such as physical activity intensities, are more accessible to users. These works motivate us to move forward and study the feasibility of directly using actigraphy collected by off-the-shelf activity trackers for ADHD diagnosis and monitoring in adolescents.

Machine learning has provided new opportunities for ADHD identification. For example, support vector machines have been used to classify ADHD in adults based on eye openness and neuropsychological tasks,^37^ altered event-related potentials,^38^ and EEG data.^39^ Random forests were used for feature selection and identification of ADHD for adults using multimodal serotonergic data.^40^ Duda *et al.*^41^ proposed to distinguish autism spectrum disorder from ADHD with patient-reported questions based on support vector machines and logistic regression. However, previous studies did not explore the use of commercial activity trackers for identifying ADHD and classifying medication status, nor did they compare the performance of different measures. Whereas previous actigraphy-based studies mainly relied on medical or research devices for data collection and usually had short data collection periods, this study applied advanced machine learning techniques to the data collected from off-the-shelf activity trackers for 24-hour continuous monitoring for 3 months, serving as a crucial first step in research development in the diagnosis and monitoring of ADHD in Chinese adolescents with commercial wearable technology, providing initial insights to guide larger and more comprehensive studies.

Although our findings have yielded significant insights, it is essential to address the limitations and consider areas for future research. To the best of our knowledge, this is the first work on studying the feasibility of using commercial activity trackers and machine learning techniques for the diagnosis and monitoring of ADHD in Chinese adolescents. Given that this is a feasibility study, the sample size was small to allow for an initial assessment. The majority of the participants were Chinese children, which may lack cultural and ethnic diversity. The data collection procedures also required the participants to have smartphones. Whereas the experimental results showed the effectiveness and reliability of the methods in the subjects of this study, the findings, such as the key features identified, may lack generalizability. Future research could aim to recruit a larger sample of participants with various backgrounds to be more representative. Specifically, the framework and techniques of our study are compatible with a larger dataset, which presents an opportunity for scaling our research methods to broader populations and more varied demographic groups. We believe our study can be a crucial first step in research development, providing essential insights that guide larger and more comprehensive studies.

Second, more subjective and objective features (eg, academic performance, financial conditions, activity types) can be incorporated to further improve the model performance and generalization, providing more insights into the important features correlated with ADHD. We believe that integrating contextual features, such as the time of day the activity occurs, the location settings (indoor vs outdoor), the nature of the activity (structured vs unstructured), and more fine-grained motion patterns not only can refine the accuracy of ADHD diagnoses but also enhance the general applicability of the models across diverse real-world scenarios. This enhancement is expected to lead to more personalized and precise health care solutions, further leveraging the advanced capabilities of machine learning in medical diagnostics.

Third, the parent-reported measures were collected years before this study was conducted. The reported ADHD symptoms may not have been accurately reflected in the participant when conducting the study. Fourth, when building the machine learning model, as XGboost is robust to the multicollinearity of features,^6^ we did not process the correlated features to retain as much information as possible. The correlation between features would influence the interpretation of models. We also recognize the potential for recall bias with WhatsApp-based self-reporting of Fitbit removal and medication usage in our study, but maintain that the platform’s widespread accessibility and familiarity with the participants for convenient data reporting likely mitigated this concern and contributed to the consistency and continuity of communication.

Moreover, as reported in the poststudy acceptance survey, the distractions caused by wearable devices and restrictions on wearable electronic device usage imposed by some schools could have impacted the popularization of wearable devices among adolescents. Despite these challenges, wearable activity trackers are becoming increasingly popular worldwide, with adolescents emerging as major users. This suggests a growing acceptance and potential benefits that may outweigh the initial hurdles. Further research could promote school acceptance and drive companies to develop more user-friendly and compact wearables. The limited transparency in data and algorithms by commercial devices due to proprietary interests remains a barrier. Advocating for standardized reporting protocols and deeper academia-industry collaborations could facilitate wider data sharing and the development of open-source algorithms, enhancing the trustworthiness and applicability of wearable technology in health care.

Future studies may also employ casual models to obtain more in-depth insight into the influence of various features and explore within-individual variance to provide insights into individual-level changes and factors influencing ADHD symptoms and treatment response, leading to more personalized interventions. Lastly, this study focused primarily on group-level analysis to identify patterns and trends across the entire sample. Although the within-individual variance was not specifically assessed in this study, it is an important aspect to consider in future research. Future studies should aim to collect recent parent-reported measures.

The implications of our findings are summarized from the clinical and societal perspectives as follows. In real-life clinical settings, the use of actigraphy data collected through wearable technologies can aid in the diagnosis and monitoring of adolescents with ADHD. The objective measures provided by actigraphy may offer a noninvasive and efficient way of identifying ADHD in a clinical population, greatly reducing patient and health system burden. Furthermore, the time-coded data collected by wearable devices allows for a fine-grained analysis of activity level changes over time, which may help to identify both interindividual and intraindividual differences in activity. This approach may be useful in identifying particularly challenging times of day and aid treatment optimization.

Our findings highlight resting heart rate, mean heart rate, and very active minutes as the key features for classifying medicated ADHD adolescents. Stimulants, commonly prescribed for ADHD, are known to increase heart rate as a side effect. Wearable technology facilitates real-time monitoring of cardiac impacts and provides insights into basal cardiac activity, potentially elevated in youths taking stimulants. Tracking these changes can help assess the cardiovascular stress these medications may impose, especially in a long-term treatment scenario. This continuous data can be crucial for detecting patterns that might require medical attention or adjustment of medication dosages. Furthermore, we observed higher active minutes in the medicated group, aligning with research suggesting stimulants affect energy levels and physical activity.^42^ Using wearable technology to monitor these parameters offers a noninvasive, continuous, and real-time method to evaluate the efficacy and safety of medication in everyday settings, enhancing personalized medication management and therapeutic outcomes. Integrating these physiological and activity metrics monitored with clinical observations enables health care providers to gain a more comprehensive view of the impact of the medication, leading to better-informed treatment decisions. This holistic approach underscores the value of wearable technology in managing complex conditions such as ADHD treated with CNS stimulants. The promising results of this feasibility study underscore the need for larger and more definitive studies to thoroughly investigate the observed effects and ensure the reliability and applicability of the results.

The increasing popularity of wearable devices, especially wrist-worn fitness trackers, has opened up new prospects in health care, making health care more accessible. By analyzing the health-related wearable signals, many health issues can be identified proactively. While developing wearable health care applications, it is important to ensure that these technologies are accessible and effective across diverse cohorts, such as groups in the minority, to prevent bias and ensure the applicability to all ethnic and socioeconomic groups. While traditional health care is often expensive and constrained by geographical and logistical challenges, wearable technology is a promising way to make health care more pervasive. However, if wearable devices should become increasingly expensive, it would exacerbate existing health inequalities. Therefore, inclusivity principles are crucial to make these devices affordable for all socioeconomic groups. Simplifying device features or using less expensive materials could reduce costs. It is also significant to safeguard privacy and data security, especially for young users, to maintain trust. Furthermore, advocating for policies that subsidize the cost of these devices and integrate them into public health strategies can promote health equity, allowing adolescents from diverse backgrounds to equally benefit from wearable ADHD monitoring technology.

In conclusion, the integration of objective activity monitoring using wearable devices and machine learning techniques shows great promise for improving the diagnosis and monitoring of ADHD in adolescents. Objective measures collected by Fitbit alone exhibit the ability to classify ADHD and ADHD medication statuses, whereas combining subjective and objective features enhances overall performance. Incorporating objective measures into clinical practice can reduce health care burden, improve diagnostic accuracy, and enhance treatment outcomes. The use of wearable devices enables noninvasive and continuous monitoring of activity patterns, supplementing existing diagnostic protocols and allowing for personalized interventions to manage ADHD symptoms.

## CRediT authorship contribution statement

**Zhihan Jiang:** Writing – review & editing, Writing – original draft, Visualization, Validation, Methodology, Formal analysis. **Adrienne Y.L. Chan:** Writing – review & editing, Writing – original draft, Validation, Investigation, Data curation, Conceptualization. **Dawn Lum:** Writing – original draft, Resources, Investigation, Data curation. **Kirstie H.T.W. Wong:** Writing – review & editing, Validation, Resources, Investigation, Data curation. **Janice C.N. Leung:** Writing – review & editing, Resources, Data curation. **Patrick Ip:** Writing – review & editing, Supervision, Project administration, Funding acquisition, Data curation. **David Coghill:** Writing – review & editing, Validation, Resources. **Rosa S. Wong:** Writing – review & editing, Resources, Investigation, Data curation. **Edith C.H. Ngai:** Writing – review & editing, Supervision, Software, Project administration, Funding acquisition, Conceptualization. **Ian C.K. Wong:** Writing – review & editing, Supervision, Resources, Project administration, Funding acquisition.
