# Supplementary material for: Wearable Signals for Diagnosing Attention-Deficit/Hyperactivity Disorder in Adolescents: A Feasibility Study
Source: JAACAP Open. 2024 Nov 25;3(4):875–89. doi: 10.1016/j.jaacop.2024.11.003 (PMC12684460; doi:10.1016/j.jaacop.2024.11.003)
Supplement: Supplement 1 [file mmc1.docx]

Supplement 1: Recruitment Process

WhatsApp invitational messages (1) with a recruitment flyer (2) and an online interest form (Supplement 2) were sent to the cohort of Chinese adolescents. A member of the research team (anonymized information) conducted eligibility screening interviews and discussed potential participation with eligible and interested families via a phone call (3). These families were sent an information statement (4) and consent form (5) and were invited to the face-to-face enrolment interview and baseline assessment at anonymized information. We provide both English and Traditional Chinese versions in this Supplement.

Table of Contents

[I. Recruitment WhatsApp Template 2](#_Toc146753320)

[II. Recruitment Flyer 3](#_Toc146753321)

[III. Phone Call Script 5](#_Toc146753322)

[IV. Parent Participant Information Letter 11](#_Toc146753323)

[V. Briefing Guide – To Participants 23](#_Toc146753324)

# **I. Recruitment WhatsApp Template**

Hello! A team of researchers anonymized information is starting a new research project. This project involves adolescents aged 12-17 years who have participated in previous studies from the anonymized information. Participants will be reimbursed $1200 and a Fitbit for their time. For more information, please visit the website: anonymized information or scan the QR code on this flyer. Thanks!

您好，

anonymized information現正進行一項新研究項目🧐📚📈，並誠邀曾參與過由anonymized information的研究，年齡介乎12至17 歲的青少年參與此項新研究項目🙇🏻‍♀️🙏🙇🏻‍♂️。為答謝參加者在研究過程中所付出的時間🙏，在研究結束時可得到$1200💰🤩💰現金並可在研究結束後保留研究團隊所提供的 Fitbit ⌚智能手錶。🙌🤩🙌

🥳 如有興趣了解更多信息，請到訪此網站👇🏻 ：anonymized information 或掃描圖片上的二維碼🤳📱。

謝謝!😊

# **II. Recruitment Flyer**

Some information in the flyer has been masked for anonymization


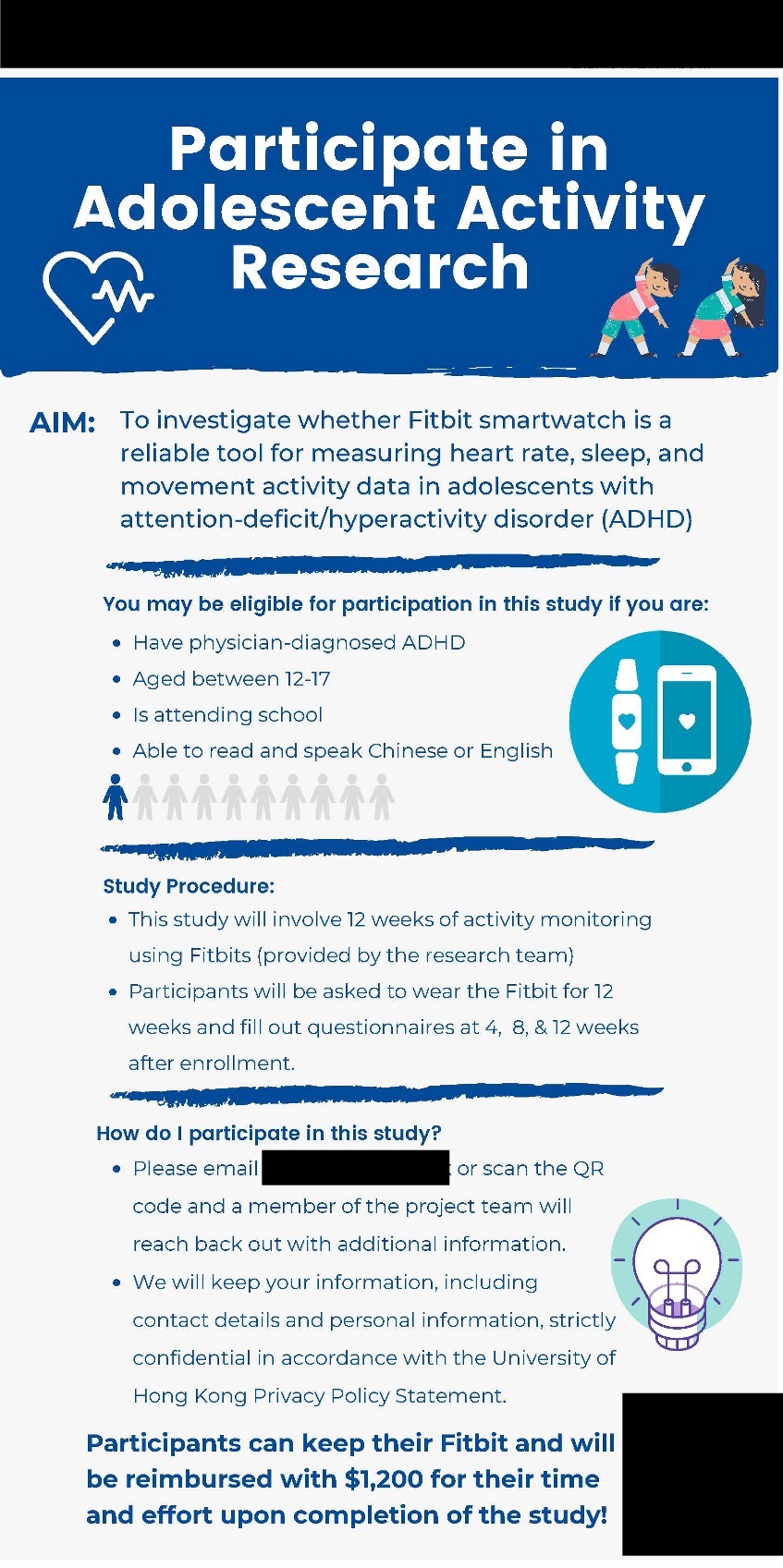


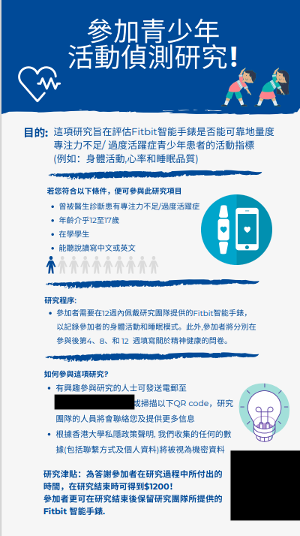


# **III. Phone Call Script**

**INTRO**

“Hi! This is [NAME] from anonymized information calling about your interest in the ADHD Fitbit Study. Is this [ INTERESTED PERSON]?”

**STUDY INFO**

No: “Sorry about that, thank you for your time!” *END CALL*

Yes: “Great! I am calling to talk about the Fitbit Activity Study you expressed interest in. Do you have time right now?”

- No: “Okay no worries. Do you have a time you’re more available? Or we can communicate through email if that is preferred.”
  - Time: “What time and date works for you? ( their response ) Okay! We will call you back then. Thanks!” *END CALL*
  - Email:” Okay! Is [THEIR EMAIL] the right one?”
    - If no, write down the right one
    - If yes: “Great we will email you there. Thanks!” *END CALL*

- Yes: “Okay! I will walk you briefly through the study and answer any questions you may have. So firstly to talk about the study, this is a study looking to see if Fitbits can be used to accurately measure heart rate, sleep, and movement data in people with ADHD. Participants will be asked to wear the Fitbit for 12 weeks and answer questions on sleep and Fitbit removal every week, as well as fill out follow-up questionnaires every 4 weeks. The research team will passively track heart rate, sleep, and physical activity data. Participants will also be asked to download the Fitbit app on their smartphones and sync the data every night. A shared Fitbit account will be provided, so that both the research team and participants can see the information during the study. All information will be kept confidential and data collected will be de-identified before analyses. After the 12 weeks, participants can log out of the research Fitbit account and keep their Fitbit, as well as get $1200 reimbursement for their time and effort. Any questions so far?”
  - Yes: *Answer their questions~*
  - No: “Okay great!” ~ *Go to next section*

**CEDI PERMISSION**

No: “After collecting Fitbit data, we would like to access your past CEDI survey data and CDARs clinical records in order to compare the data we receive from the Fitbit with past medical records and treatment you’ve received for ADHD. We hope that by comparing the data, we will improve real world monitoring of ADHD symptoms, as well as treatment effectiveness and responsiveness in order to help improve future diagnosis and treatment for adolescents with ADHD. Would you be willing to allow the research team access to these records?”

- - Yes: “Great! On the consent form, there is an opt in box for the CEDI data. Please check the box before you submit the form. Thank you! “ ~ *Go to next section*
  - No: “That’s okay! Unfortunately, you will be unable to participate in this study as this is a crucial part. However, while you may be unable to participate in this study, if there are any studies that interest you in the future, please feel free to reach out. Thank you again for your time!” **End**

**ENROLLMENT**

“Would you be interested in enrolling in our study?”

- Yes: “Great! Firstly, could I have the participant’s name? (Write down name on excel) We would like to schedule a time for you and your child to pick up the Fitbit and do the baseline questionnaires as well. You will also receive the initial $300 of the reimbursement during this visit. Would you like to schedule the appointment now?
  - Yes: *~schedule appointment and move on to next section*
  - No: “No worries! We will send over more information on how to schedule the Fitbit pickup date in an email after this phone call. Please sign up in your own time!” *~move to next section*
- No: “Okay thank you for your time! Please feel free to reach out if you have any other questions and have a nice day.“

**FOLLOW UP:**

- “Perfect thank you!
  - *IF TALKING TO GUARDIAN:* **Please make sure to bring the signed consent form, a mobile phone to sync to the fitbit and your child’s HKID on the arranged date to pick up the Fitbit.**
  - *IF TALKING TO TEEN:* **(Please make sure your guardian signs the consent form before the arranged pick up date.)**
- The signed consent form can either be emailed back anonymized information or you can print it and give it to us on the day you pick up your Fitbit. Does this arrangement sound okay to you?”
  - Yes:~*Move onto next section*
  - No: *Help them then move to last section*

**END**

“All right, that’s about it from us. Would you also like us to explain the study to your (guardian/ child) as well?

- Yes: Ok! Can you please provide their contact information and a time we can call them?
- No: **Move on*

Any questions before we end this call?”

- No: “Great! Then have a nice day and we will be in touch via email shortly.”
- Yes: *Answer them until they’re good to go!*

*-END-*

介紹

“您好！我係anonymized information嘅 [NAME]，我見到你對我地嘅ADHD Fitbit研究項目有興趣，想了解多啲你嘅情況。請問係唔係______?”

Add in project name – be more specific

研究項目信息

不是：“對不起，多謝你嘅時間！” *結束通話*

是：“你好呀！我地打黎係想同你傾番你之前表示會有興趣參加既Fitbit 研究。請問你而家有無時間？”

没有：“唔緊要。請問你有咩時間方便我可以再打黎? 或者我地都可以用電郵聯絡”

時間: “幾點會方便你？ （他們的回答）好呀！咁到我遲啲再打黎啦。唔該哂！” *結束通話*

電子郵件: 好嘅！請問 [THEIR EMAIL] 係唔係你嘅email？

如果不是，請寫下正確的

如果是：“OK! 我地會將研究簡介電郵俾你。唔該哂！” *結束通話*

是的：“我而家同你簡單介紹番呢項研究並解答番您可能遇到的任何問題。首先這項研究是想了解Fitbits 可唔可以準確地測量 ADHD 患者的心率、睡眠和運動數據。參與者會需要戴住 Fitbit 12 個星期，並喺每 4個星期填寫一次有關睡眠同除下Fitbit嘅時間嘅問卷。研究團隊將以被動形式收集參與者嘅心率、睡眠和身體活動數據。參與者需要喺手提電話度下載Fitbit嘅手機程式，而且每晚需要進行一次數據同步。我們將提供一個Fitbit 帳戶俾參與者，以便研究團隊和參與者在研究過程中都可以看到信息。所有信息都將保密。 12 個星期後，參與者可以登出研究 Fitbit 帳戶並保留他們的 Fitbit，並獲得 一共1200 元的研究津貼。到目前為止請問有無問題或者唔明白既地方？”

有：回答他們的問題~

o 没有：“好，我地落下一個環節” ~ 進入下一節

CEDI 允許在收集 Fitbit 數據後，我地希望了解您過去的健康新一代研究和 醫管局 臨床記錄，以便將我地可以同 Fitbit數據做個比較。通過這個比較希望可以改善現時醫生、家長觀察 ADHD 症狀的方式，同治療有效性和反應。請問你願意讓研究團隊接觸這些記錄嗎？”

同意：“請在提交表格前在同意書上，可以選擇同意允許研究團隊使用健康新一代研究 數據。謝謝！ “ ~ 進入下一節

不同意：“唔緊要，但因為呢個係我地研究既關鍵部分, 所以很抱歉你唔可以參與我地呢項研究。雖然您可能無法參與這項研究，但如果您將來有任何感興趣的研究，歡迎你隨時與我們聯繫。再次感謝您的時間！” *完*

註冊

“你有興趣參加我們的研究嗎？”

有：我地完咗呢個電話後會通過電郵發送同意書。我地想同你個約個時間黎anonymized information領取 Fitbit 並進行基線問卷調查，同時你都會得到港幣$300作爲參與研究的首次津貼。請問您會想而家約埋定係稍後再約？

是：~約時間並進入下一部分

不：“不用擔心！我們完咗呢個電話會後通過電子郵件發送有關如何安排領取 Fitbit的詳情。到時請您揀選合適的時間報名！” ~移至下一節

不：無問題，如果您有任何其他問題請隨時與我們聯繫。謝謝你的時間！

跟進

好嘅，唔該晒！

如果同監護人講：請確保喺領取fitbit當日前簽好份同意書

如果同參與者講：請確保你嘅監護人喺領取fitbit當日前簽好份同意書

“謝謝！請在領取 Fitbit之前簽好同意書。(If talking to teen, the GUARDIAN needs to sign consent form) 已簽署的同意書可以通過電郵事先提交，或可以打印出來喺領取 Fitbit當天交給我們。請問呢個安排有無問題？”

冇問題：~進入下一部分

有問題：幫助他們然後進入最後一部分

結尾

“介紹呢個研究項目就大概講到咁多啦，你會唔會想我地解釋埋比你嘅（監護人/小朋友）聽？”

想：可以呀，麻煩你寫低

唔想：*落下一條問題

“請問對於我地呢項研究你仲會唔會有其他問題想問？”

- 冇問題：“多謝你對我地研究既支持，我地將很快通過電子郵件與您聯繫。”

- 有問題：回答他們，直到他們沒有問題

| **IV. Parent Participant Information Letter** | | | |  |  |
| --- | --- | --- | --- | --- | --- |
| **Research Project Title:** | Real-time Activity Monitoring Study | | | |  |
| **Principal Researcher:** | anonymized information | | | | |
| **Version Number:** | 1 | **Version Date:** | 4/06/202021 | | |

Thank you for taking the time to read this Parent/Guardian Information Statement and Consent Form. We would like to invite your child to participate in a research project that is explained below. This document is 5 pages long. Please make sure you have all the pages.

**What is an Information Statement?**

These pages tell you about the research project. It explains to you clearly and openly all the steps and procedures of the project. The information is to help you decide whether or not you would like your child to take part in the research. Please read this Information Statement carefully.

Before you decide if you want your child to take part or not, you can ask us any questions you have about the project. You may want to talk about the project with your family, friends or health care worker.

If you would like your child to take part in the research project, please sign the consent form at the end of this information statement. By signing the consent form you are telling us that you:

- understand what you have read
- had a chance to ask questions and received satisfactory answers
- consent to your child taking part in the project.

We will give you a copy of this information and consent form to keep.

1. **What is the research project about?**

| This research project is a pilot study to investigate whether Fitbit smartwatch is a reliable tool for measuring heart rate, sleep, and movement data in people with [attention-deficit/hyperactivity disorder](https://www.mayoclinic.org/diseases-conditions/adult-adhd/symptoms-causes/syc-20350878) (ADHD). We are going to measure 2 different kinds of activity, these include: 1) physical activity, this will be measured by body movement and 2) body functioning activity, this will be measured by heart rate and sleep. We will measure these different kinds of activity to see which ones are most useful, acceptable and helpful at identifying and managing difficulties. We hope that this understanding will lead us to more objective and time efficient ways of observing patterns in day to day life. |
| --- |

1. **Who is funding this research project?**

| This research is funded by the anonymized information. |
| --- |

1. **Why is my child being asked to be in this research project?**

| We are asking you and your child to take part in this project because your child is aged between 12 and 17 years, and are a participant of previous research projects led by the Principal Researcher (anonymized information). |
| --- |

1. **What does participation in this research involve?**

| Participation involves your child wearing a Fitbit provided by the research team for 12 weeks that records heart rate, physical activity and sleep patterns.  Participation in the study will involve an initial face-to-face visit with you and your child that will take approximately 90 minutes. This visit will take place at anonymized information.  This visit involves:   - Fitting your child with a Fitbit, going through the safe use guide and downloading the Fitbit app onto your child’s phone to link to the Fitbit for syncing purposes. The research team will set up a Fitbit account and will provide account login details. Account details will not contain any information that can identify your child. - You and your child completing some surveys.   As well as this first visit the study includes follow up surveys after 4 weeks, 8 weeks and 12 weeks.  If you agree to participate in this study we will also need to access your child’s HealthyKids electronic medical record and CDARS clinical records to obtain information about any prescribed medication and/or treatment during the study period. If you consent, there will be option to opt in in the consent form below.  Throughout the study the research team will monitor your child’s Fitbit syncing via the research Fitbit account. If a member of the research team identifies any issues with syncing or that no data has been synced for a period of 4 consecutive days they will send an SMS to you or your child with instructions and a reminder to sync.  One week after the initial visit a member of the study team will call you to check in and see if there are any issues with the research technology. You will also be provided with details to contact the study team if your child experiences any issues with the research technology throughout the study.  **Optional consent**  We would also like to seek your consent for re-contacting you about other research in the future.  In the future, new research projects may be suitable for you and your child. We would like your permission to contact you about these projects. We will give you information about new projects then. It is up to you whether or not you agree to take part in a future project. |
| --- |

1. **What are my child’s alternatives to taking part?**

| Participation in a research project is voluntary. Your child does not have to take part in this project if they do not want to or if you do not want them to.  You and your child can change your minds and withdraw from the study at any time without giving a reason. If your child withdraws from the study we will arrange a study closure visit to return the Fitbit. We will ask your permission to use any information already collected, if you do not agree to this then we will destroy that information.  Your decision will not affect any treatment or care your child gets, or your family’s relationship with anonymized information. |
| --- |

1. **What are the possible benefits for my child and other people in the future?**

| As well as earning **$100 per week** of participating upon completion, we expect the main benefits of this study to be for others in the future. We hope the information we get will lead to a better understanding of every day activity patterns of young people with mental health difficulties, which may lead to improved diagnosis and treatments. |
| --- |

1. **What are the possible risks, side-effects, discomforts and/or inconveniences?**

| During the surveys we will be asking you and your child questions about sensitive topics. If you or your child feels stressed or anxious about any of the questions, these questions do not have to be answered. The information you and your child provide to us during the surveys will be kept confidential, but in certain circumstances we may be required to disclose something you or your child have told us to help keep you and your family safe. This would only occur if you or your child were to tell us something that makes us seriously worried about your safety or the safety of your child. If this happens, a member of the research team would discuss his or her concerns with you before seeking additional support for you or your child.  If you or your child become upset or distressed as a result of your participation in the research project, the research team can arrange for appropriate support. This may include linking you with support services for you, or informing the anonymized information for your child.  During the trial your child will wear a research provided Fitbit on their wrist continuously for 12 weeks. If your child experiences any signs of skin irritation or wrist rash we ask that they remove the Fitbit at the first sign and that you inform a member of the research team. The research team will instruct your child to stop wearing the Fitbit for 2-3 days. If skin irritation resolves, your child will be allowed to resume wearing the Fitbit. If irritation does not resolve or reoccurs your child will be instructed to stop wearing the Fitbit and inform the research team. The research team will direct you to consult a GP about your child’s skin condition, withdraw your child from the study and arrange a study closure visit to return the Fitbit. |
| --- |

1. **What will be done to make sure my child’s information is confidential?**

| In this study we will collect and use personal and health information about your child for research purposes. Any information we collect that can identify your child will be treated as confidential and used only in this project unless otherwise specified. We can disclose the information only with your permission, except as required by law. If a member of the research team is concerned about you or your child’s safety, they will need to share this with a clinician on the research team to ensure the appropriate support is provided.  All survey and Fitbit information will be stored securely at anonymized information.  The following people may access information collected as part of this research project:   - the research team involved with this project - anonymized information   The stored information will be re-identifiable. This means that we will remove identifying information such as your child’s name and give the information a special code number. Only the research team can match your child’s name to their code number, if it is necessary to do so.  We are required to keep information collected as part of a research project for a certain length of time. Because the participants in this project are under 18 years old, we must keep information until a participant turns 25 years old. The research information may be destroyed or kept indefinitely in secure storage after this time.  In accordance with relevant Hong Kong privacy and other relevant laws, you have the right to access and correct the information we collect and store about your child. Please contact us if you would like to access this information.  At the end of the study, results may be presented at conferences or published in medical journals. This will be done in such a way that your child cannot be identified. |
| --- |

1. **Will we be informed of the results when the research project is finished?**

| We will send you a summary of the study results at the end of this project. This summary will give overall results of this project – individual families will not be identified. |
| --- |

If you would like more information about the project or if you need to speak to a member of the research team in an emergency please contact:

| **Name:** | anonymized information |
| --- | --- |
|  |  |
| **Email** | anonymized information |

**Confidentiality:** The right of access to personal data and publicly available study results exists, if and when needed. Under the laws of Hong Kong (in particular the Personal Data (Privacy) Ordinance, Cap 486), rights for the protection of the confidentiality of personal data exist, such as those regarding the collection, custody, retention, management, control, use (including analysis or comparison), transfer in or out of Hong Kong, non-disclosure, erasure and/or in any way dealing with or disposing of any of your personal data in or for this study. For any query, please consult the Privacy Commissioner for Personal Data or his office (Tel No. 2827 2827) as to the proper monitoring or supervision of personal data protection so that full awareness and understanding of the significance of compliance with the law governing privacy data is assured.

**Upon consent**, you agree that 1) The principal investigator and his research team and the corresponding ethics committee responsible for overseeing this study are authorized to get access to, to use, and to retain personal data for the purposes and in the manner described in this informed consent process; and 2) The relevant Hong Kong Special Administrative Region government agencies (e.g., the Department of Health and Hospital Authority) are authorized to get access to personal data for the purposes of checking and verifying the integrity of study data and assessing compliance with the study protocol and relevant requirements.

**CONSENT FORM**

| **HK IRB Number:** | anonymized information | | |  |
| --- | --- | --- | --- | --- |
| **Research Project Title:** | Real-time Activity Monitoring Study | | | |
| **Version Number:** | 1 | **Version Date:** | 4/06/2021 | |

- I have read, or had read to me in my first language, the information statement version listed above and I understand its contents.
- I believe I understand the purpose, extent and possible risks of my child’s involvement in this project.
- I voluntarily consent for my child to take part in this research project.
- I have had an opportunity to ask questions and I am satisfied with the answers I have received.
- I understand that this project has been approved by anonymized information Human Research Ethics Committee.
- I understand I will receive a copy of this Information Statement and Consent Form.

| YES, I do | NO, I do not | voluntarily consent for me and my child to take part in the above research project |
| --- | --- | --- |
| YES, I do | NO, I do not | Consent to researchers accessing my child’s previous CDARS and “HealthyKids” data for this study’s ADHD data linkage |
| YES, I do | NO, I do not | consent to the researchers uploading the results of my child’s assessment to their electronic medical record |

OPTIONAL CONSENT

| YES, I do | NO, I do not | consent to re-contact for future research |
| --- | --- | --- |

| Child’s Name |  |  |  |  |
| --- | --- | --- | --- | --- |

| Parent/Guardian Name |  | Parent/Guardian Signature |  | Date |
| --- | --- | --- | --- | --- |

Note: All parties signing the Consent Form must date their own signature.

| ­ | **致參加者家長信函** | | |  |  |
| --- | --- | --- | --- | --- | --- |
| **研究項目名稱:** | 實時活動監測研究Real-time Activity Monitoring Study | | | |  |
| **首席研究員:** | anonymized information | | | | |
| **版本編號:** | 1 | **版本日期:** | 4/06/2021 | | |

感謝您抽空閱讀這份家長/監護人聲明及同意書。我們誠邀您的子女參與以下的研究項目。這份信函共有5頁，請確定頁數齊全。

**這份是什麼信函？**

這信函旨在介紹這項研究計劃的詳情，包括此項目的所有步驟和程序。希望能幫助您決定是否參加這項研究。請仔細閱讀本聲明。

您可以在決定是否參與前向我們查詢任何有關此研究項目的資料。您也可以跟家人，朋友或醫護人員討論此項目。

如果您同意讓您的子女參加這研究項目，請在此信函最後一頁的同意書上簽署。簽署同意書即表示您：

- 已閱讀及了解本信函的內容
- 已有機會提出問題並獲得滿意的答覆
- 同意您的子女參與此研究項目

我們將向您提供這份資料和同意書副本，以便保存。

1. **這是一項關於什麼的研究項目？**

| 這是一項先導研究項目, 研究的主要目的是了解Fitbit智能手錶能否可靠地量度[專注力不足/ 過度活躍症](https://www.dhcas.gov.hk/file/conditions/ADHD_Chi_1706.pdf)患者的心率，睡眠和活動數據，以作爲專注力不足/過度活躍症的指標。.我們將量度兩種不同的活動，包括1）將透過身體活動2）身體功能活動－將透過量度心率和睡眠情況。 我們期望透過量度各類型的活動，以了解最有效和合適的方法來識別和管理病人日常所遇到的困難。 希望幫助我們日後更客觀和更有效率地觀察青少年的日常活動。 |
| --- |

1. **這研究項目由誰資助？**

| anonymized information。 |
| --- |

1. **為什麼我的子女會被邀請參與這個研究項目？**

| 由於您的子女年齡介乎12至17歲，而且曾參加過以往由anonymized information領導的研究項目，因此我們誠邀您和您的兒女參與。 |
| --- |

1. **若參與這項研究****，需要完成什麼測試項目？**

| 在同一參與研究後，您的子女需要在之後的12週佩戴著研究團隊提供的Fitbit智能手錶，以記錄您子女的身體活動, 心率,和睡眠模式。  另外，本團隊也會邀請您和您的子女進行約90分鐘的初步面談訪問。此訪問將在香港大學進行。  訪談内容包括：   - 介紹如何使用Fitbit，閱讀安全使用指南，以及將Fitbit應用程序下載到他/她的手機上並連接到Fitbit作同步運作。我們會為他/她設立一個Fitbit賬戶，並提供登入賬戶的詳情。賬戶內並不會包含任何可以識別您的子女身份的個人資料。 - 您和您的子女完成問卷調查。     除了第一次會面外，另外也需要完成三次的問卷調查分別會在在第四周，第八周和第十二周進行。  如果您同意您的子女參與本研究項目，我們希望讀取您的子女的CEDI電子病歷和CDARS臨床記錄，以獲取有關研究期間任何處方藥物和/或治療的資料。若同意，可在一下的同意書中選擇加入。  在整個研究過程中，本團隊將通過研究中使用的Fitbit帳戶監察您子女的Fitbit同步運作的數據。 如果我們發現同步運作的數據有任何問題，或者連續4天未有同步更新到任何數據，則會向您或您的子女發送一條簡訊，其中包含進行同步運作的指示和提醒。  首次訪談的一週後，研究團隊的人員會致電給您以查看Fitbit或研究應用程序會否出現任何問題。我們也會向您提供研究團隊的聯絡方式，以便您的子女在研究過程中遇到技術問題時可聯繫我們。  **自願同意書**  為了方便將來有適合您和您的子女的新研究項目時再次聯繫您，我們希望徵求您的同意，以便將來就這些項目再次與您聯繫。我們到時候會提供有關新項目的詳情以及同意書，而最終是否同意參加新研究項目也取決於到時候您的決定。 |
| --- |

1. **我的子女還有其他什麼選擇？**

| 參與研究項目是自願性的。 如果您或您的子女不想參加此項目，則不必參與。  您和您的子女亦可以隨時改變主意並退出研究，決定退出時無需給予理由。 如果您的子女選擇退出研究，我們將安排時間讓您將Fitbit交還給本團隊。 我們將徵求您的許可來使用已收集的資料，如果您不同意，我們將銷毀那些資料。  您的決定不會影響您的子女從anonymized information獲得的任何治療或護理，也不會影響anonymized information與您和您的家人之間的關係。 |
| --- |

1. **這研究對我的子女和其他人可以帶來什麼好處？**

| 若您的子女完成了這項研究，除了研究期間每週賺取$ 100外，我們主要期望望這項研究能在日後給其他人帶來益處。 我們希望得出的資料可以令人更深入了解有精神障礙的年輕人的日常活動規律，從而可改善診斷和治療方法。 |
| --- |

1. **有哪些潛在的風險、副作用、不適和/或不便？**

| 在問卷查當中，某些問題可能較爲敏感。如果您或您的子女對任何問題感到有壓力或焦慮，則不必回答。您和您的子女在問卷調查中提供的資料將被保密，本團隊只會在涉及您或您的子女安全的情況底下才允許研究人員得知有關您的資料，以保障您和您的子女的安全。如果發生這種情況，研究人員將先與您們討論您的子女的憂慮，然後再幫您們尋求解決方案。  如果您或您的子女因參加研究項目而感到困擾或苦惱，研究團隊可為您們安排適當的支援。這可能包括為您的子女通知anonymized information。    在研究過程中，您的子女需要連續12週在手腕上佩戴研究用的Fitbit。如果您的子女的皮膚上出現任何皮疹或發紅，或者因佩戴Fitbit而感到疼痛或不舒服，請他/她立即除下Fitbit，並通知我們的研究人員。我們會提議他/她停止佩戴Fitbit 2-3天。如果皮疹/發紅褪去，他/她可以繼續佩戴Fitbit。如果皮疹/紅腫沒有褪去或再次出現，請他/她停止佩戴Fitbit，並通知我們的研究人員。我們將轉介您的子女就手腕皮疹/發紅去就醫。您的子女將需要退出研究項目，我們亦會為您們安排交還Fitbit的時間。 |
| --- |

1. **如何確保我的子女的資料是保密的？**

| 在本研究中，我們會以研究目的爲由來收集和使用有關您的子女的個人和健康資料。 除非另有說明，否則任何可以識別您子女身份的信息將被視為機密資料，僅在本項目中使用。 除法律要求外，我們只會在您的允許下披露資料。 如果研究人員對您或您的子女的安全感到擔憂，則有需要與研究小組的醫生分享，以確保您們獲得適當的幫助。  所有問卷調查和Fitbit資料將安全地儲存於anonymized information。  以下人員可讀取此研究項目中收集的資料：   - 參與該項研究的研究團隊 - anonymized information研究操守委員會   儲存的資料將可被重新識別。意思就是我們將刪除某些可被識別的信息，例如：姓名等，並為該資料提供特殊的代碼。如有必要，只有研究團隊才能以代碼去相識別出他/她的姓名。  我們需要將收集到的資料作為研究項目的一部分保存一定的時間。由於此研究項目的參加者不到18歲，因此我們必須保留記錄，直到參加者年滿25歲。此後，研究信息可被銷毀或無限期保存在安全的存儲器中。  根據香港和其他相關法律，您有權閱讀和更正我們收集和儲存有關您子女的資料。如果您想閱讀您子女的個人資料，請與我們聯繫。  在研究結束時，研究結果有機會在會議或在醫學期刊上發表。任何在發表文獻中提及到有關參加者的資料將不會被識別到。 |
| --- |

1. **研究項目結束後，我們會否獲知結果？**

| 在研究結束時，我們將向您發送研究結果的摘要。 此摘要將提供此項目的總體結果，並會將個人身份或家庭資料調整到無法被識別的模式。 |
| --- |

如果您想了解更多有關該研究項目的資料，或者在緊急情況下需要與研究團隊的人員聯絡，請聯繫：

| **姓名:** | anonymized information |
| --- | --- |
|  |  |
| **電郵：** | anonymized information |

**個人資料保密：**如有需要，每位研究參與者都有權利獲得其個人資料以及公開報告中的研究結果。根據香港法律（特別是「個人資料（隱私）條例」第486章），您有保護個人資料機密性的權利，例如在本項研究中或與本研究有關的個人資料的收集、保管、保留、管理、控制、使用（包括分析或比較），在香港內外轉讓，保密，刪除和/或以任何方式處理。如有任何疑問，請諮詢香港個人資料私隱專員公署或致電到其辦公室（電話號碼：2827 2827），以適當監管或監督您的個人資料保護，並確保此研究項目當中收集到的資料會根據和遵守保護私隱資料及遵守相關法律的方式儲存。

**若您同意****的話**，則表示您同意1）授權主研究人員及其研究團隊和負責監督本研究的倫理委員會獲得，使用和保留用於本研究的個人資料，按照您的子女知情同意底下所説明的程序和方式進行；和2）授權香港特別行政區相關政府機構（例如衛生署和醫院管理局）讀取個人資料，以檢查和驗證此研究的廉正以及對研究方案的合規評估及相關要求。

**同意書**

| **HK IRB項目編號:** | anonymized information | | |  |
| --- | --- | --- | --- | --- |
| **研究項目名稱:** | 實時活動監察研究 | | | |
| **版本編號:** | 1 | **版本日期:** | 4/06/2021 | |

- 我已閱讀上列的資料聲明，並且理解其內容。
- 我相信我了解我的子女參與此研究的目的，範圍和潛在的風險。
- 我自願同意我的子女參與這個研究項目。
- 我已有機會提出問題並獲得滿意的答覆。
- 我了解這研究項目已獲得anonymized研究操守委員會的批准。
- 我了解我將收到此資料聲明和同意書的副本。

| 我同意 | 我不同意 | 同意我和我的子女參與以上研究項目。 |
| --- | --- | --- |
| 我同意 | 我不同意 | 研究人員訪問我孩子以前的「健康新一代」I 和CDARS臨床記錄數據以用於此注意力不足／過度活躍症研究的數據鏈接 |
| 我同意 | 我不同意 | 研究人員將我子女的評估結果上載至他/她的電子病歷中。 |

同意書

| 我同意 | 我不同意 | 你們為將來的研究再次聯繫我。 |
| --- | --- | --- |

| 參加者姓名 |  |  |  |  |
| --- | --- | --- | --- | --- |

| 家長/監護人姓名 |  | 家長/監護人簽署 |  | 日期 |
| --- | --- | --- | --- | --- |

注意：簽署同意書時必須使用自己的簽名

# **V. Briefing Guide – To Participants**

Outline for when participants are here

1. Make sure Fitbits are logged in and ready (charged)
2. Consent form and info sheet run through again – make sure to collect signatures
3. Then fill out baseline surveys
4. Give Fitbit – run through (briefing) (they also need to download app)
   1. Expectation for syncing
5. Set expectations for number of questionnaires and when we’ll give it to them (as well as reason why)
6. Give them the $300 and make sure they provide signature and HKID

Check w/ reception to see how they receive guests!

Checklist Before Participant Has Arrived:

*Make sure Fitbit account info is ready and Fitbit charged*

*Ipad is set up with the surveys*

*Info Sheet and Consent Forms ready*

*$300 cash*

Script:

**CONSENT FORM/ INFO SHEET**

RA: First we’ll briefly go over the information sheet and consent form. Did you have a chance to look over either of them?

- Yes: Okay! Would you like me to go over it again?
  - Yes: **Go to NO section explaining survey*
  - No: **move onto next section**
- No: (*Take out study calendar)* Okay no worries. So this study is looking to investigate whether the Fitbit is a reliable tool for measuring heart rate, sleep and movement data in people with ADHD. You will be wearing a Fitbit provided by the research team for 12 weeks that records heart rate, physical activity, and sleep patterns. The Fitbit account will be provided by our team and you will be provided login details. In addition to wearing the Fitbit, we will be asking you to download the Fitbit app + sync the Fitbit. We will also be asking some sleep questions once a week and if there were points in the week where you took off the Fitbit for more than an hour. Furthermore, follow up questionnaires will be sent out on week 4, week 8, and week 12 in the study. After the study is over, we will do a data correlation between the activity seen on the Fitbit and the participant’s 健康新一代 (HealthyKids) records and CDARS clinical records. We hope to use this information to see if the Fitbit can be a reliable clinical tool for people with ADHD in the future. For your participation, we will be gifting the Fitbit after the study, as well as $1,200 total.
- Then the last page is the Consent form. Please sign if you’re okay with what the study will entail or let us know if we can answer any questions for you. **answer any questions they may have!*

**BASELINE SURVEYS**

Let’s fill out the initial set of questionnaires now. You will be prompted to fill out these questionnaires every 4 weeks. They will be sent to you over email. *Is there a preferred email you would like these questionnaires to be sent to?* **(mark down email)**

| Participant | Email / Whatsapp (EMAIL PREFERRED) |
| --- | --- |
|  |  |
|  |  |
|  |  |
|  |  |
|  |  |

- ***If no email, mark down their whatsapp number*** *and we will whatsapp the anon link to each survey to fill out every 4 weeks.*

1. Demographic (baseline survey) anonymized information
2. TEXI anonymized information
3. PEDSQL anonymized information
4. CES-D anonymized information
5. ARI anonymized information

In addition to these 4 surveys, we will also be asking you every Monday about times you’ve taken off the Fitbit throughout the past week and also a few sleep questions once a week. We will only be asking about your sleep the night before and the questions will be asked on a random day each week. The reason the questions will be asked on a random day is because we want to ensure the Fitbit data quality by cross checking the sleep data we see on the Fitbit to what you experienced.

**FITBIT**

**Give them Fitbit*

Here’s the Fitbit you will be using. We have a anonymized information account login set up for you to use.

Please also download the Fitbit app on your phone now. **help them download and login if needed**

Google Play (Android) Apple Store


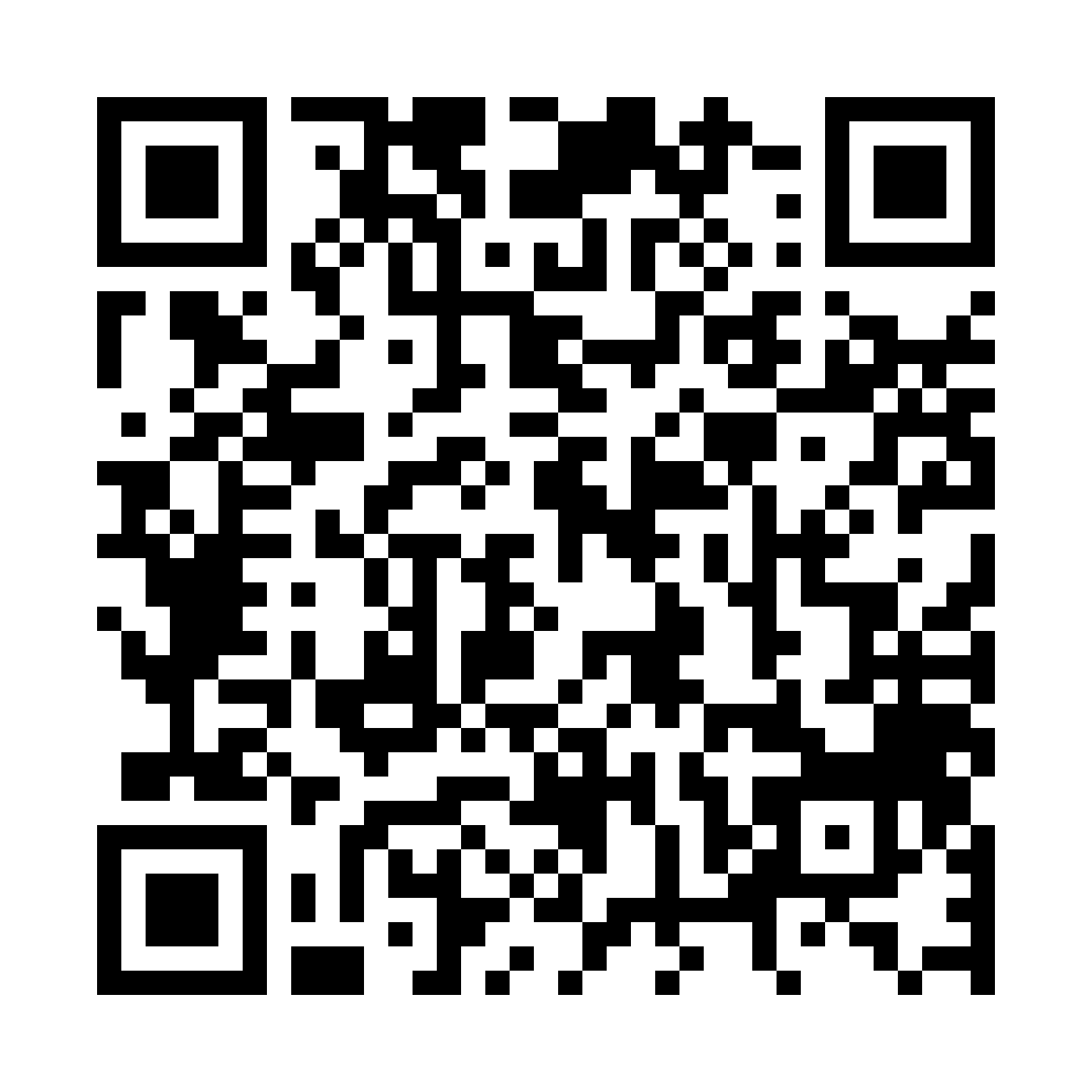

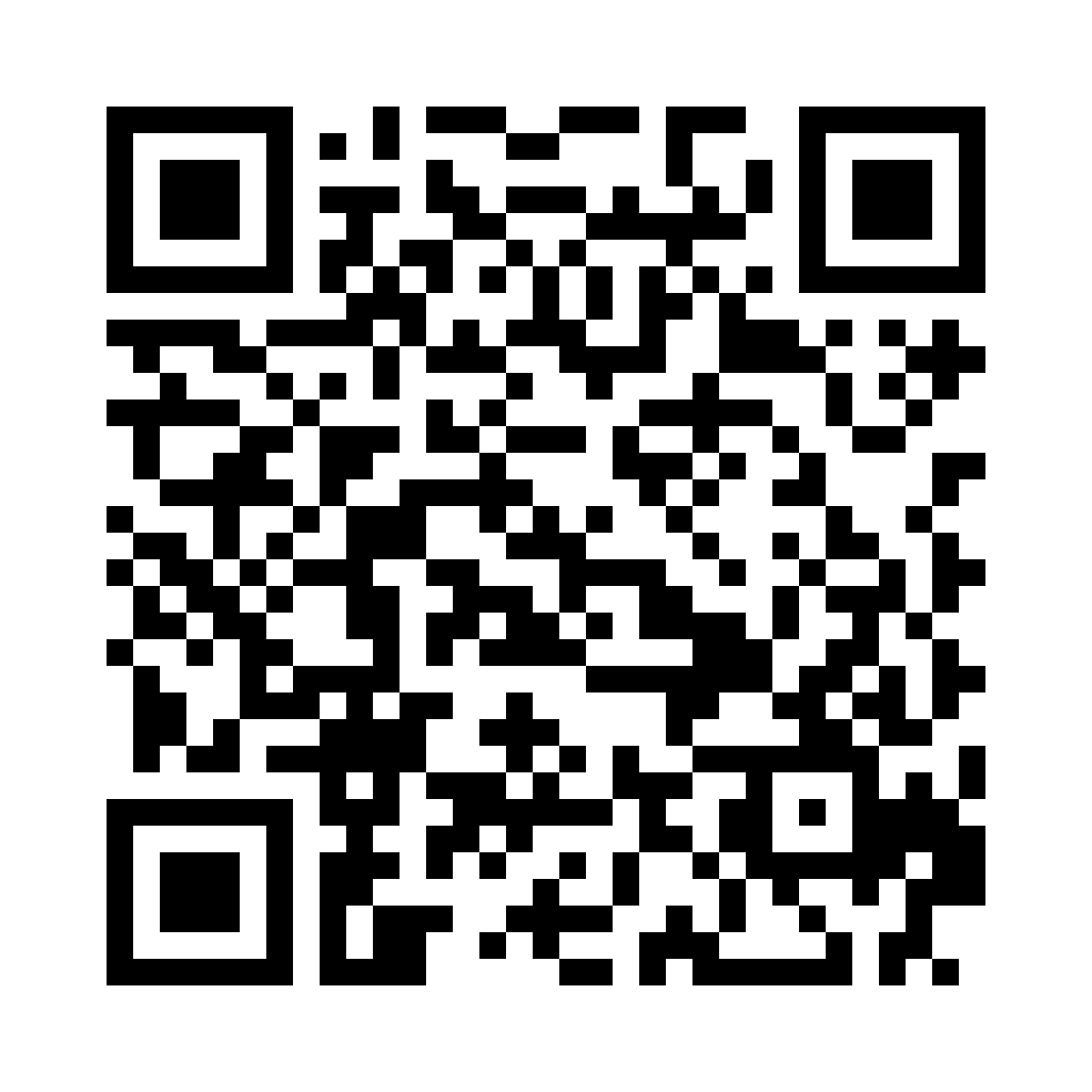


Please log into the Fitbit app with the anonymized information Login (Provide Email and PW for participants)

- We used a generic birthday, weight and height. Please modify the weight and height yourself in your own time (Home screen -> Top left icon -> Click View Your Profile -> Personal -> Edit and Save

Then Connect your Charge 4 to the account. Go to Home screen -> Top left icon -> Set Up a Device -> Charge 4 -> Follow Instructions (location/ GPS tracking optional)

*Using Fitbit*

Click the button on the left to go to home screen or go “back”. You can swipe Right to connect to Spotify, settings, etc. If you swipe up from the Home Screen, you can see your Step count, heart rate, etc

Please wear the Fitbit at all times except if it will get wet (eg. Shower, swim).

If you experience discomfort or irritation, try to loosen the band, and if it continues, please take of the device for a day and email a anonymized information team member to let them know.

*Keeping it Clean*

- Fitbit is water resistant but not waterproof. Please don’t wear it when showering or swimming!
- To clean the Fitbit band, please ONLY use water and soap-free cleanser (eg. Cetaphil) (not soap or alcohol)

*Sync*

Please sync the Fitbit every night. The Fitbit needs to be synced in order for us to access the data. The Fitbit app is automatically set to “all-day sync” but this may drain your battery. You can turn it off (Profile -> Charge 4 -> Scroll down on device settings) and the Fitbit automatically syncs once the Fitbit app is open but may take a few minutes to finish syncing. You can also press “sync now” **show participants syncing*

If we see that the Fitbit data hasn’t synced, we may contact you as a gentle reminder. Do you have a preference of means of contact (Call or Whatsapp)?

**Write down means of contact**

| Participant | Call or Whatsapp for Sync Reminder |
| --- | --- |
|  |  |
|  |  |
|  |  |
|  |  |
|  |  |

We will contact you first, but if there’s no reply within a reasonable amount of time, we may also contact your guardian to remind you to sync the data. This data is obviously extremely important for this study so please make sure you sync the Fitbit regularly.

*Charging*

To charge the Fitbit, disconnect the Fitbit from the band by pressing both buttons at the same time underneath the Fitbit. Then clamp the Fitbit into the Fitbit charger (matching the metal on the charger to metal on the Fitbit). Plug in charger and Fitbit should be charging!

When Fitbit needs to charge, try charging while you’re showering as you will not be wearing it anyways!

Any questions? **Answer any questions they may have*

**REIMBURSEMENT GENERAL RULES**

You will be reimbursed $100 every 7 days for 12 weeks ($1,200 total) and you will be able to keep the Fitbit at the end of the study.

Deductions in reimbursement per week **may** occur for either of the following ($50 per missed requirement): 1. If more than 3 days of Fitbit activity is skipped in a week 2. The required follow up questions / questionnaires are not completed for the week.

Therefore, if 3+ days of data is missed and follow up questionnaires aren’t complete, there **may not be** reimbursement for that week.

Reimbursement will be given in 2 stages. In good faith, $300 will be given in advance today. At the end of the study, we will schedule the participant to come back to anonymized information to collect their remaining reimbursement ($900 without deductions).

Any questions?

**answer questions they may have*

That’s about it from us. So just a general reminder, please wear the Fitbit at all times except when it may get soaked (eg. Showering, swimming) and charge when needed. Follow up Questionnaire links will be emailed to you every 4 weeks (week 4, 8, 12) and we will be asking you to fill out some sleep and Fitbit wearing questions once a week as well. If you have any questions or concerns, please email anonymized information

**INITIAL $**

Finally, along with the Fitbit, we will be giving you the initial $300 of the reimbursement in good faith. Please sign this Qualtrics form with the first 4 characters of your HKID and signature in order to receive it.

- **Have participants sign Qualtrics form* ***(Make sure their HKID format is correct!! (A123 or XA123))***
- anonymized information
- END -
