# Supplementary material for: Wearable Signals for Diagnosing Attention-Deficit/Hyperactivity Disorder in Adolescents: A Feasibility Study
Source: JAACAP Open. 2024 Nov 25;3(4):875–89. doi: 10.1016/j.jaacop.2024.11.003 (PMC12684460; doi:10.1016/j.jaacop.2024.11.003)
Supplement: Supplement 2 [file mmc2.pdf]

English

RSVP/Signup Form

Hello and thank you for your interest in the Real-time Activity Study. Please fill out this interest form, upon completion of this form, you will receive a document on study details. Once the study begins (tentatively early/mid September), a researcher on the team will reach out again to schedule a time to sign the consent form and pick up the Fitbit at (anonymized information) .If you have any questions, please email (anonymized information) .

您好，感謝您對Real-time Activity Study 的興趣。填寫完此電子表格後，將通過電郵向您發送一份關於本研究的更詳細的家長信息表。研究開始後 (暫定九月初或中旬)，團隊中的一名研究人員將再次聯繫以簽署同意書，並安排時間在(anonymized information) 領取 Fitbit。如有任何疑問，請電郵至(anonymized information) 。

A team of researchers at (anonymized information)is conducting a pilot studyto determine whether it is feasible and helpful to monitor activity in adolescents aged 12-17. We wish to learn whether we can use Fitbits to gain a better understanding patterns of heart rate, physical activity, and sleep patterns in everyday life.

ADHD Diagnosis

Are you the participant or the participant’s parent/guardian?

- ○ Participant
- ○ Participant's parent/guardian

Please provide the following contact information.

Name (First and Last) [ ]

Email

Phone Number

Has the participant been diagnosed with ADHD?

- Yes
- No

Unfortunately we are only including people diagnosed with ADHD in our current study. However, would you be interested in being contacted once this study expands to future participant pools?

- Yes
- No

Does the participant take medication for ADHD?

- Yes
- No

Is the participant currently on drug holiday?

- Yes
- No

When will the participant begin taking medication again? (What month or date)

screenout

Has the participant experienced psychosis in the past 2 weeks, been clinically diagnosed with Autism, experience epilepsy or have Tourette's?

- Yes
- No

Does the participant have any other major illness or disability including neurological and movement disorders that may confound the actigraphy data?

- Yes, please specify: ____
- No

Block 2

Did you learn this research study from (anonymized information) team?

- Yes
- No

After the study is over, we will do a data correlation between the activity seen on the Fitbit, the participant's 健康新一代 (HealthyKids) records, and the CDARS clinical records. We hope to use this information to see if the Fitbit can be a reliable clinical tool for people with ADHD in the future. Will you agree to let our research team access your clinical and past survey data?

- Yes
- No

Unfortunately we are only including people in "HealthyKids" in our current study. However, would you be interested in being contacted once this study expands to future participant pools?

- Yes

○ No

Please enter the participant's year of birth (to ensure they're within the study age range)

Powered by Qualtrics
