# Supplementary material for: Wearable Signals for Diagnosing Attention-Deficit/Hyperactivity Disorder in Adolescents: A Feasibility Study
Source: JAACAP Open. 2024 Nov 25;3(4):875–89. doi: 10.1016/j.jaacop.2024.11.003 (PMC12684460; doi:10.1016/j.jaacop.2024.11.003)
Supplement: Supplement 3 [file mmc3.docx]

Supplement 3: Details of Metrics and SHAP Values Calculations

Table of Contents

[I. The definitions and calculations of each model performance evaluation metrics 1](#_Toc146754204)

[II. The calculations of SHAP values 2](#_Toc146754205)

# **I. The definitions and calculations of each model performance evaluation metrics**

The AUC score is calculated by varying the probability threshold from 0.0 to 1.0 with an increment of 0.01 and getting the corresponding true positive rate and false positive rate to estimate the area under the ROC curve. Based on the ROC curves, we select the optimal probability threshold achieving a high true positive rate (TPR) and low false positive rate (FPR) based on the Youden Index, i.e., selecting the threshold maximizing TPR-FPR. The values of accuracy, sensitivity, specificity, positive predictive value, and negative predictive value on the optimal threshold were calculated for each participant group, as shown in Equation (1). Accuracy was the proportion of true results among the total number of cases examined. Sensitivity was the proportion of case-positive participant data that were predicted as positives by the ML model. Specificity was the proportion of case-negative participant data that were predicted as negatives by the model. Precision, or positive predictive value, was the probability of predicting participants as cases. Sensitivity, specificity, positive predictive value (PPV), accuracy, and area under the receiver operating characteristic curve (AUC) were used as performance indexes.

$Sensitivity=\frac{TP}{TP+FN}, Specificity=\frac{TN}{TN+FP}, PPV=\frac{TP}{TP+FP},NPV=\frac{TN}{TN+FN}$, (1)

where TP is the true positive instance. TN is the true negative instance. FN is the false negative instances, and FP is the false positive instances.

# **II. The calculations of SHAP values**

SHAP is a game-theoretic method that computes the contribution of each feature in a machine-learning model. It can be implemented efficiently, especially for tree-based models. In this study, SHAP analysis was used to understand the “global” impact of input features on the overall model. The SHAP values also provide the capability to explore the “local” feature effects, which illustrates the impact of input features on individual predictions. More specifically, suppose $X=\left\{ \left( x_{i}, y_{i} \right) \right|i=1,2,\ldots, N\}$ is the training set, where $x_{i}$ and $y_{i}$ are the input features and output of participant i, and N is the number of participants in the training set. Then, the output $y_{i}$ follows the equation below:

$y_{i}=E\left[ f\left( X \right) \right]+\sum_{j=1}^{M} f\left( x_{ij} \right),i=1,2,\ldots,N,$ (2)

where E[f(X)] is the baseline value of the model, which is the expected output of the model (usually the mean of the predicted value of all training samples), and $f\left( x_{ij} \right)$ is the SHAP value of $x_{ij}$, meaning the contribution of the $j^{th}$ feature of $i^{th}$ sample to the output $y_{i}$. Therefore, if $f\left( x_{ij} \right)>0$, it means that the $j^{th}$ feature of $i^{th}$ sample increases the output of $y_{i}$, and vice versa.
