# Supplementary material for: Wearable Signals for Diagnosing Attention-Deficit/Hyperactivity Disorder in Adolescents: A Feasibility Study
Source: JAACAP Open. 2024 Nov 25;3(4):875–89. doi: 10.1016/j.jaacop.2024.11.003 (PMC12684460; doi:10.1016/j.jaacop.2024.11.003)
Supplement: Supplement 4 [file mmc4.docx]

Table S1: Feasibility of applying Fitbit technology to routine monitoring or diagnosis of attention-deficit/hyperactivity disorder (ADHD).

|  | **ADHD** | | **W/o ADHD** | |
| --- | --- | --- | --- | --- |
| n | **13** | | **14** | |
| **Study instruction clarity:** |  |  |  |  |
| Couldn't understand at all | 0 | 0% | 0 | 0% |
| Slightly Unclear | 0 | 0% | 0 | 0% |
| Neutral | 2 | 15% | 1 | 7% |
| Slightly Clear | 6 | 46% | 6 | 43% |
| Very Clear, Easy to Understand | 5 | 38% | 8 | 57% |
|  |  |  |  |  |
| **Problems while wearing Fitbit: (Multiple selection)** |  |  |  |  |
| Uncomfortable | 7 | 54% | 6 | 43% |
| Distracting | 5 | 38% | 5 | 36% |
| Easy to forget to re-wear | 7 | 54% | 11 | 79% |
| Inconvenient | 6 | 46% | 4 | 29% |
| Other | 2 | 15% | 4 | 29% |
|  |  |  |  |  |
| **Impact on lifestyle outside of school** |  |  |  |  |
| Very negatively impacted | 0 | 0% | 0 | 0% |
| Somewhat negatively impacted | 0 | 0% | 0 | 0% |
| No impact | 8 | 62% | 10 | 71% |
| Somewhat positively impacted | 3 | 23% | 3 | 21% |
| Very positively impacted | 2 | 15% | 1 | 7% |
|  |  |  |  |  |
| **Impact on school life** |  |  |  |  |
| Very negatively impacted | 0 | 0% | 0 | 0% |
| Somewhat negatively impacted | 3 | 23% | 1 | 7% |
| No impact | 8 | 62% | 11 | 79% |
| Somewhat positively impacted | 1 | 8% | 1 | 7% |
| Very positively impacted | 1 | 8% | 1 | 7% |
|  |  |  |  |  |
| **Continue using Fitbit** |  |  |  |  |
| Definitely not | 0 | 0% | 0 | 0% |
| Probably not | 0 | 0% | 1 | 7% |
| Might or might not | 4 | 31% | 4 | 29% |
| Probably yes | 4 | 31% | 6 | 43% |
| Definitely yes | 4 | 31% | 2 | 14% |

**Note:** ADHD = attention-deficit/hyperactivity disorder; W/o ADHD = without ADHD.

Table S2: Rankings of features based on SHAP (SHapley Additive exPlanation) values.

| **Features** | **Ranking based on absolute SHAP values for classifying ADHD and w/o ADHD** | **Ranking based on absolute SHAP values for classifying medicated and unmedicated ADHD** |
| --- | --- | --- |
| SWAN Hyperactivity Score | 1 | 21 |
| ARI Total Score | 2 | 16 |
| PedsQL Physical Health | 3 | 4 |
| Sex | 4 | 18 |
| Distance | 5 | 7 |
| Calories Burned | 6 | 20 |
| SWAN Inattentive Score | 7 | 13 |
| TEXI Working Memory | 8 | 6 |
| Sleep Time | 9 | 17 |
| Lightly Active Minutes | 10 | 23 |
| Resting Heart Rate | 11 | 1 |
| Maximal Heart Rate | 12 | 24 |
| CES-D Total Score | 13 | 10 |
| PedsQL Total Score | 14 | 15 |
| TEXI Total Score | 15 | 12 |
| TEXI Inhibition | 16 | 11 |
| PedsQL Psychosocial Health | 17 | 14 |
| SWAN Total Score | 18 | 25 |
| Sedentary Minutes | 19 | 22 |
| Fairly Active Minutes | 20 | 8 |
| Very Active Minutes | 21 | 3 |
| Activity Calories | 22 | 19 |
| Hourly Steps | 23 | 9 |
| Mean Heart Rate | 24 | 2 |
| Floors | 25 | 5 |

**Note:** The SHAP values are from the model trained on objective, self-reported subjective, and parent-reported features for classifying attention-deficit/hyperactivity disorder (ADHD) and without (w/o) ADHD and classifying medicated and unmedicated ADHD. The table is sorted on the ranking of absolute SHAP values for classifying ADHD and w/o ADHD. ARI = Affective Reactivity Index; CES-D = Center for Epidemiologic Studies Depression Scale; PedsQL = Pediatric Quality of Life Scale; SWAN = Strengths and Weakness of ADHD Symptoms and Normal Behavior Scale; TEXI = Teenage Executive Functioning Inventory Scale.
